# Aging human abdominal subcutaneous white adipose tissue at single cell resolution

**DOI:** 10.1111/acel.14287

**Published:** 2024-08-14

**Authors:** K. L. Whytock, A. Divoux, Y. Sun, M. F. Pino, G. Yu, C. A. Jin, J. J. Robino, A. Plekhanov, O. Varlamov, S. R. Smith, M. J. Walsh, L. M. Sparks

**Affiliations:** ^1^ Translational Research Institute, AdventHealth Orlando Florida USA; ^2^ Icahn School of Medicine at Mount Sinai New York City New York USA; ^3^ Department of Genetics, School of Medicine Stanford University Stanford California USA; ^4^ Divisions of Metabolic Health and Disease Oregon National Primate Research Center Beaverton Oregon USA

**Keywords:** aging, senescence, single nuclei RNA‐Seq, White adipose tissue

## Abstract

White adipose tissue (WAT) is a robust energy storage and endocrine organ critical for maintaining metabolic health as we age. Our aim was to identify cell‐specific transcriptional aberrations that occur in WAT with aging. We leveraged full‐length snRNA‐Seq and histology to characterize the cellular landscape of human abdominal subcutaneous WAT in a prospective cohort of 10 younger (≤30 years) and 10 older individuals (≥65 years) balanced for sex and body mass index (BMI). The older group had greater cholesterol, very‐low‐density lipoprotein, triglycerides, thyroid stimulating hormone, and aspartate transaminase compared to the younger group (*p* < 0.05). We highlight that aging WAT is associated with adipocyte hypertrophy, increased proportions of lipid‐associated macrophages and mast cells, an upregulation of immune responses linked to fibrosis in pre‐adipocyte, adipocyte, and vascular populations, and highlight CXCL14 as a biomarker of these processes. We show that older WAT has elevated levels of senescence marker p16 in adipocytes and identify the adipocyte subpopulation driving this senescence profile. We confirm that these transcriptional and phenotypical changes occur without overt fibrosis and in older individuals that have comparable WAT insulin sensitivity to the younger individuals.

## Graphical abstract

This study performed full‐length snRNA‐Seq and histological analyses on white adipose tissue (WAT) from 10 younger and 10 older individuals split evenly for sex and balanced for body mass index. We highlight that aging WAT is associated with adipocyte hypertrophy, increased proportions of lipid‐associated macrophages and mast cells, and an upregulation of genes related to immune responses. We show that older WAT has elevated levels of senescence marker p16 in adipocytes and identify the adipocyte subpopulation driving this senescence profile.

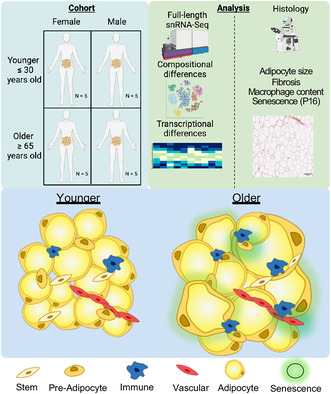

**Abbreviations**
ADIPO‐IR: Adipose Tissue Insulin Resistance Index
ARI: Adjusted Rand Index
BMI: Body Mass Index
CRP: C‐Reactive Protein
DEGs: Differentially Expressed Genes
ECM: Extracellular matrix
FDR: False Discovery Rate
FFA: Free‐Fatty Acids
LAM: Lipid‐Associated Macrophages
LISI: Local Inverse Simpson’s Index
snRNA‐Seq: single‐nuclei RNA‐sequencing
TRI: Translational Research Institute
UMAP: Uniform Manifold Approximation and Projection for Dimension Reduction
WAT: White Adipose Tissue
WHR: Waist‐to‐Hip Ratio

## 1 INTRODUCTION

Aging is associated with a progressive decline in physiological function leading to augmented human pathology and vulnerability to death (López‐Otín et al., 2013). White adipose tissue (WAT) functions as a robust energy store, an endocrine organ that governs whole‐body metabolic homeostasis, and a tissue that regulates immune modulation and regeneration (Goodpaster & Sparks, 2017; Palmer & Kirkland, 2016). Impairments in WAT function lead to unfavorable WAT redistribution towards central abdominal stores (Kuk et al., 2009), ectopic lipid accumulation and subsequent peripheral insulin resistance in organs such as skeletal muscle and liver (Borén et al., 2013; Shulman, 2014), and low‐grade chronic systemic inflammation (Starr et al., 2009; Wu et al., 2007). Elevated cellular senescence (Justice et al., 2018; Tchkonia et al., 2010; Xu, Palmer, et al., 2015), reduced progenitor proliferation, impaired adipogenic potential of the progenitor pool (Caso et al., 2013), and immune cell infiltration (Trim et al., 2022) have all been purported as factors contributing to age‐associated decline in WAT function. These prior analyses, however, have largely been restricted to in vitro assessments or targeted histological and FACS approaches, and there is extremely limited human data. Single cell/nuclei and spatial transcriptomics in human subcutaneous WAT is a rapidly growing area of research (Acosta et al., 2017; Angueira et al., 2021; Bäckdahl et al., 2021; Divoux et al., 2024; Emont et al., 2022; Hildreth et al., 2021; Jaitin et al., 2019; Karunakaran et al., 2020; Massier et al., 2023; Merrick et al., 2019; Sun et al., 2020; Vijay et al., 2020; Whytock et al., 2022; Ye et al., 2024; Zhou et al., 2023) with the effects of aging on the cross‐talk between progenitor and immune cells recently being explored in a limited scope (Zhou et al., 2023).

Our aim was to provide a comprehensive and untargeted assessment of the compositional and nuclei‐type‐specific transcriptional changes that occur in aging WAT. In this study, we prospectively obtained abdominal subcutaneous WAT biopsies for single‐nuclei RNA sequencing (snRNA‐Seq) analyses from individuals who were not undergoing surgery for a pre‐existing condition. We leveraged our recent advancements in full‐length snRNA‐Seq in human WAT (Whytock et al., 2022, 2023), which yields superior gene detection capabilities compared to prior single‐cell/nuclei RNA‐Seq platforms and technologies such as 10X Genomics, combined with histological assessments, to assess the effects of aging on human WAT.

## 2 RESULTS

### 2.1 Physiological characteristics

**FIGURE 1:**
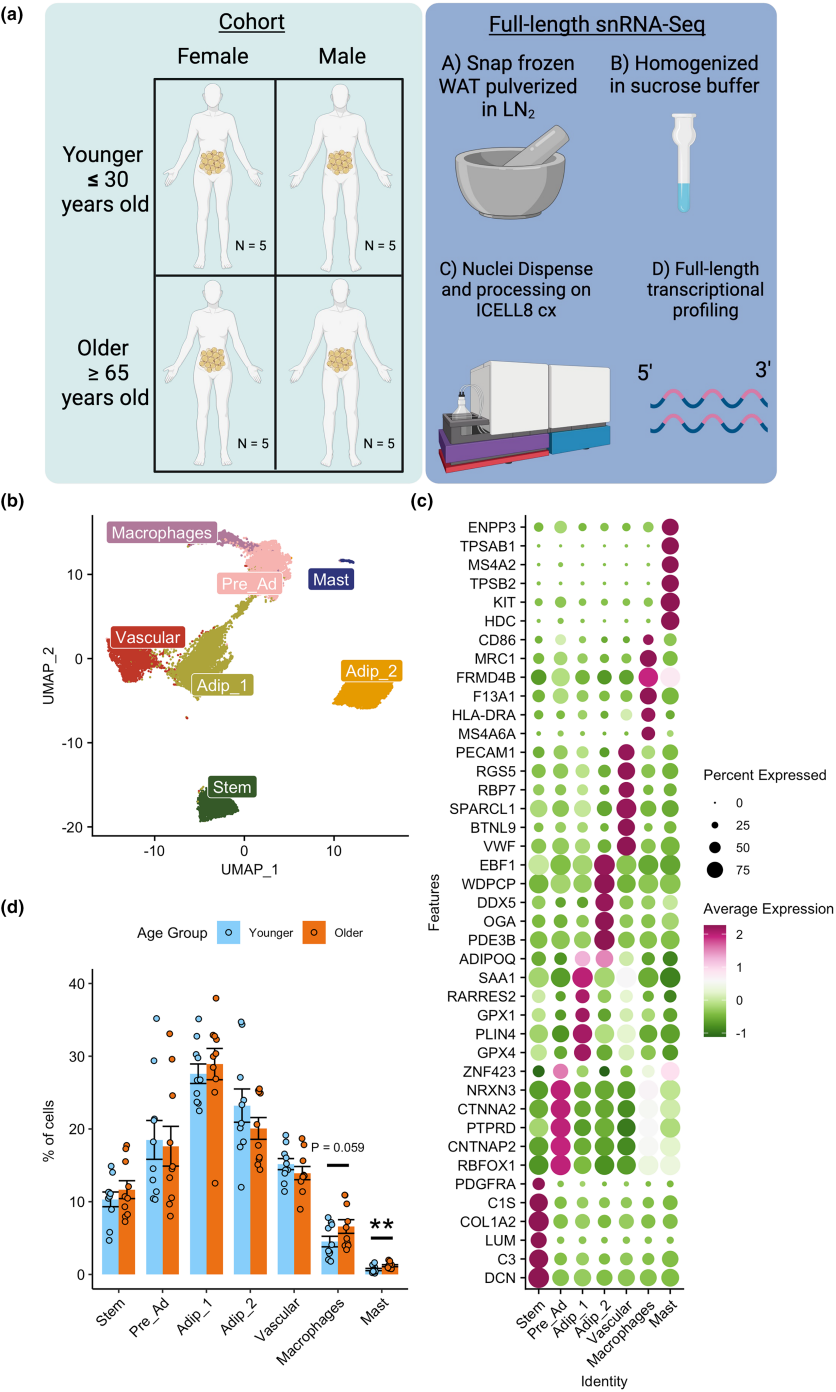
Single nuclei transcriptional profiling of aging human abdominal subcutaneous white adipose tissue (WAT). Schematic overview of study design and methods (a). UMAP of 23,702 nuclei from abdominal subcutaneous white adipose tissue (WAT) of 10 younger and 10 older participants highlighting different cell populations (b). Dotplot of top 5 upregulated differentially expressed genes for each cell population and known cell markers (*PDGFRA –* Stem, *ZNF423 –* pre‐adipocytes, *ADIPOQ –* adipocytes, *PECAM1 –* vascular, *CD86 –* macrophages, *ENPP3 – mast cells*) (c). Nuclei composition differences between older and younger participants highlighting immune cells are elevated in the older Group (d).

To explore the effects of aging on WAT, we performed full‐length snRNA‐Seq analysis on abdominal subcutaneous WAT from 10 older (≥65 years old) and 10 younger (≤30 years old) individuals balanced for sex and body mass index (BMI) (Figure 1a). See Table S1 for full participant characteristics. WAT biopsies were performed at the Translational Research Institute (TRI) (see methods for processing details). The older group had elevated systolic blood pressure, waist circumference, waist‐to‐hip ratio, plasma cholesterol, very‐low‐density lipoprotein, triglycerides, thyroid stimulating hormone, and aspartate transaminase compared to the younger group (*p* < 0.05; Table 1). There were no differences in BMI, plasma glucose, plasma free fatty acids (FFAs), serum insulin, serum C‐reative protein (CRP), nor WAT insulin sensitivity measured by ADIPO‐IR (Groop et al., 1989; Søndergaard et al., 2017) between the older and younger groups.

**TABLE 1:** Participant characteristics.

Clinical variable	Older	Younger	*p* Value
Age (years)	74 ± 7	26 ± 3	1.19E^−10^
Sex (F/M)	5/5	5/5	
Race (WH/BL/AI/UNK)	(8/1/0/1)	(7/2/1/0)	
Ethnicity (NH/H/UNK)	(5/1/4)	(2/5/3)	
BMI (kg/m^2^)	29.38 ± 4.78	28.31 ± 4.80	0.623
Waist (cm)	102.69 ± 13.39	90.81 ± 8.07	0.0272
Waist‐to‐hip ratio	0.96 ± 0.11	0.86 ± 0.08	0.0252
SBP (mmHg)	136 ± 13	122 ± 9	0.0256
DBP (mmHg)	78 ± 8	72 ± 7	0.109
Glucose (mg/dL)	97.50 ± 15.71	89.39 ± 7.23	0.151
Insulin (μIU/mL)	13.52 ± 7.36	11.34 ± 6.20	0.497
HbA1c (%)	5.63 ± 0.27	5.26 ± 0.54	0.0694
Cholesterol (mg/dL)	187.90 ± 43.51	151.20 ± 26.37	0.0349
LDL (mg/dL)	111.30 ± 42.58	80.60 ± 26.68	0.0692
VLDL (mg/dL)	21.10 ± 8.35	13.20 ± 6.32	0.0406
HDL (mg/dL)	55.50 ± 11.32	57.40 ± 12.20	0.722
Non‐HDL cholesterol (mg/dL)	132.40 ± 44.76	93.80 ± 24.53	0.0279
Triglycerides (mg/dL)	104.90 ± 41.95	66.10 ± 31.80	0.0316
FFA (mmol/L)	0.26 ± 0.14	0.34 ± 0.20	0.374
ADIPO‐IR	0.56 ± 0.33	0.69 ± 0.52	0.522
TSH (μIU/mL)	2.59 ± 1.19	1.52 ± 0.78	0.0279
ALT (units/L)	20.00 ± 3.13	26.90 ± 28.47	0.361
AST (units/L)	26.00 ± 2.16	25.70 ± 19.56	0.0278
CRP (mg/L)	2.43 ± 1.61	2.68 ± 2.82	1

Abbreviations: ADIPO‐IR, Adipose Tissue Insulin Resistance Index; AI, American Indian; ALT, Alanine Transaminase; AST, Aspartate Aminotransferase; BL, Black; BMI, Body Mass Index; CRP, C‐Reactive Protein; DBP, Diastolic Blood Pressure; F, Female; FFA, Free‐fatty Acid; H, Hispanic; HbA1c, Hemoglobin A1C; HDL, High‐Density Lipoprotein; LDL, Low‐Density Lipoprotein; M, Male; NH, Non‐Hispanic; SBP, Systolic Blood Pressure; TSH, Thyroid Stimulating Hormone; UNK, unknown; WH, White.

### 2.2 Aging human WAT single nuclei atlas

Nuclei were isolated from whole snap‐frozen WAT to explore the cellular transcriptional landscape of aging in all cell types, including adipocytes, which are typically removed with single‐cell RNA‐Seq analysis of WAT (Whytock et al., 2022). Our dataset included 23,702 nuclei initially resolved to 7 different clusters, 2 of which were adipocyte clusters (Figure 1b), with an average of 5009 genes detected per nuclei. Differentially expressed genes (DEGs) and known marker genes were used to annotate the cell clusters (Table S2). The dotplot shows the top 5 DEGs for each cluster alongside a known marker gene for each cluster; *PDGFRA* for mesenchymal stem cells, *ZNF423* for pre‐adipocytes, *ADIPOQ* for adipocytes, *PECAM1* for vascular cells, *CD86* for macrophages, and *ENPP3* for mast cells (Figure 1c). All cell clusters highly expressed known cell markers, for example mature adipocytes expressed *ADIPOQ* (Scherer et al., 1995), mesenchymal stem cells expressed *PDGFRA* (Farahani & Xaymardan, 2015), pre‐adipocytes expressed *ZNF423* (Gupta et al., 2010), vascular cells expressed *PECAM1* (Woodfin et al., 2007), macrophages expressed *MRC1* (Rőszer, 2015) and mast cells expressed *KIT* (Irani et al., 1992) (Table S2).

**FIGURE 2:**
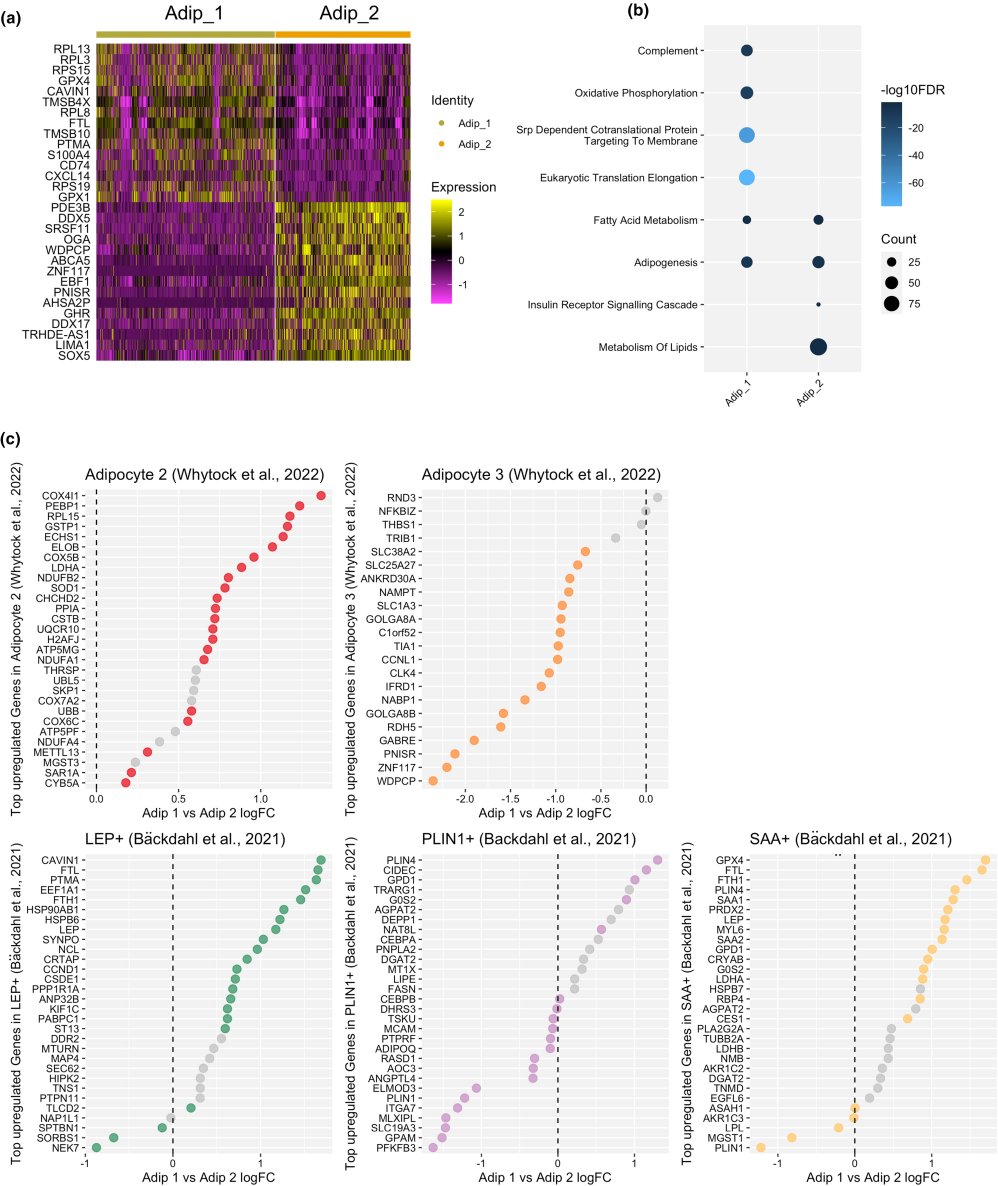
Adipocyte heterogeneity. Heatmap of top differentially expressed genes between Adip_1 and Adip_2 (a). Selected significant (FDR < 0.05) pathways upregulated in Adip_1 and Adip_2 determined by an overrepresentation test from significant (adjusted *p* value < 0.05) upregulated (logFC >0.25) genes, highlighting Adip_1 to be enriched in genes related to oxidation and immune responses whereas Adip_2 was enriched in genes related to insulin signaling and lipid metabolism (b). Comparison of differentially expressed genes from previous publications, Whytock et al. (2022) and Bäckdahl et al. (2021) in relation to differences between Adip_1 and Adip_2 highlighting transcriptional similarities from previous work (c). If the dot is colored, it signifies the gene is significantly differentially regulated between Adip_1 and Adip_2.

Due to the adipocyte clusters showing unique upregulated DEGs (Figure 1c), we further explored the differences between these two populations. When comparing Adip_2 to Adip_1, Adip_2 had an upregulation of genes related to suppression of lipolysis (*PDE3B*) and lipid metabolism (*ABCA5*), whereas Adip_1 had an upregulation of genes related to anti‐oxidation (*GPX1 & GPX4*) and ribosomal subunits (*RPL13 & RPL3*) (Figure 2a). This was represented by pathway analysis, which highlighted Adip_2 having an upregulation of pathways related to metabolism of lipids and insulin receptor signaling cascade (Figure 2b), whereas Adip_1 had upregulation of complement, oxidative phosphorylation and Srp‐dependent translational protein targeting to membrane pathways. These findings mirrored our previous characterization of mature adipocytes in snRNA‐Seq from frozen WAT (Whytock et al., 2022). To validate further, we compared the top DEGs in adipocyte clusters from our previous work (Whytock et al., 2022) and from adipocyte clusters identified using spatial transcriptomics by Bäckdahl et al. (2021). The top DEGs in adipocyte populations from our previous work completely aligned with the two adipocyte populations generated in this dataset (Figure 2c). Our Adip_2 population showed similar upregulated genes to PLIN1+ adipocytes highlighted by Bäckdahl et al. (2021), which was defined as healthy insulin‐responsive adipocytes (Figure 2c). Adip_1 shared similar upregulated genes with both LEP+ and SAA+ adipocytes from Bäckdahl et al. (2021) which may be expected given LEP+ and SAA+ adipocytes shared similarity in upregulated genes, for example, *FTL*, *FTH1*, *LEP*. Based on the transcriptional profiles alone, we have labeled Adip_1 as anti‐oxidative and Adip_2 as insulin‐responsive.

By transcriptionally profiling the nuclei isolated from WAT, we determined cell composition of adipocytes and non‐adipocyte cells using an array of markers that are not limited to pre‐specified cell markers. The largest cell type proportion in WAT is adipocytes (~50%) followed by pre‐ads (~18%), vascular cells (~15%), stem cells (~15%), and immune cells (~7%) (Figure 1d). Each participant had every cell type quantified (Figure S2A). The only compositional differences between groups were that the older group had a greater proportion of mast cells and a trend towards a greater proportion of macrophages compared to the younger group (Figure 1d). There were no differences between sexes (Figure S2B). There are different types of macrophages present in WAT (Russo & Lumeng, 2018), and therefore we sought to further define our macrophage populations. We subclustered our macrophages into two distinct clusters (Figure S3A) that had expression of resident/M2 macrophages (*F13A1*, *NAV2*) or lipid‐associated/M1 macrophages [LAMs] (*LIPA*, *TREM2*, Figure S3B) (Florance & Ramasubbu, 2022; Jaitin et al., 2019; Kim et al., 2022; Strieder‐Barboza et al., 2022; Wang et al., 2021). The increased proportion of macrophages in the older group was driven by a greater proportion of LAMs compared to the younger group (Figure S3C).

### 2.3 Cell‐type transcriptional differences between older and younger groups

**FIGURE 3:**
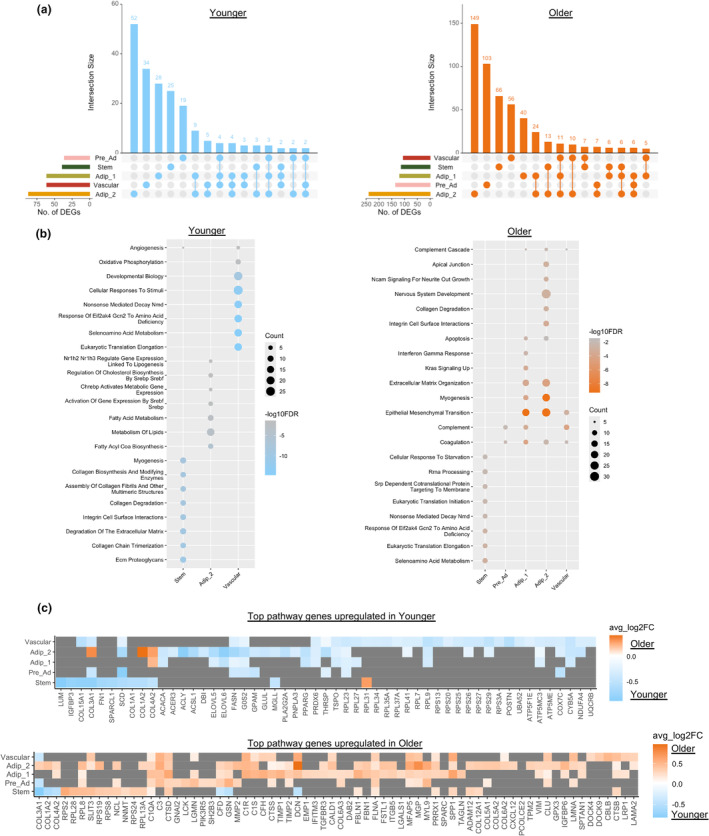
Cell‐type transcriptional differences between older and younger participants. Upset plots showing overlap of differentially expressed genes (DEGs) (adjusted *p* value < 0.05 and logFC > 0.25) between cell types that are upregulated in younger and older groups (a). Top Hallmark and Reactome pathways significantly (FDR < 0.05) upregulated in each cell type for the younger and older group determined by an overrepresentation test on significantly (adjusted *p* value < 0.05) upregulated (logFC > 0.25) genes highlighting cell‐type transcriptional differences between the groups (b). If a cell type is not present, it did not have a significant pathway upregulated. Heatmaps of top differentially expressed genes contributing to the top pathways upregulated in the younger (upper panel) and older (lower panel) groups (c). Tiles that are grey do not have gene expression significantly different between older and younger groups for that cell type.

We next sought to determine the transcriptional cellular landscape of aging WAT. Differential gene expression analysis revealed the older group had a greater number of total upregulated genes (adjusted *p* value < 0.05 and logFC > d0.25) (Figure 3a,b, Table S3). There were very few DEGs between older and younger for the macrophage and mast cell populations (Table S3) and are therefore not included in Figure 3a. Adip_2 was the most transcriptionally different cell type between younger and older as determined by the highest number of DEGs. While each cell type had a substantial number of unique DEGs between the younger and older groups, there were also shared common differentially expressed genes among different cell types (Figure 3a). Pathway analysis revealed the younger group had distinct upregulated pathways in cell types; Stem, Adip_2 (insulin responsive) and vascular (Figure 3b, Table S4) related to extracellular matrix ECM matrix, lipid metabolism, and angiogenesis, respectively. In contrast, the older group displayed common upregulated pathways among several cell types; Pre_Ad, Adip_1 (anti‐oxidative), Adip_2 (insulin responsive) and Vascular that were related to immune responses including; complement, apoptosis, and interferon gamma response (Figure 3b). Adip_2 has a transcriptional profile of an insulin‐responsive adipocyte primed for lipid storage and growth. This phenotype is exemplified in the younger group with upregulated genes related to lipid metabolism (Figure 3b, Table S5); however, the older group displays dysregulation of this process and instead displays a phenotype of inflammation.

### 2.4 Fibrosis

**FIGURE 4:**
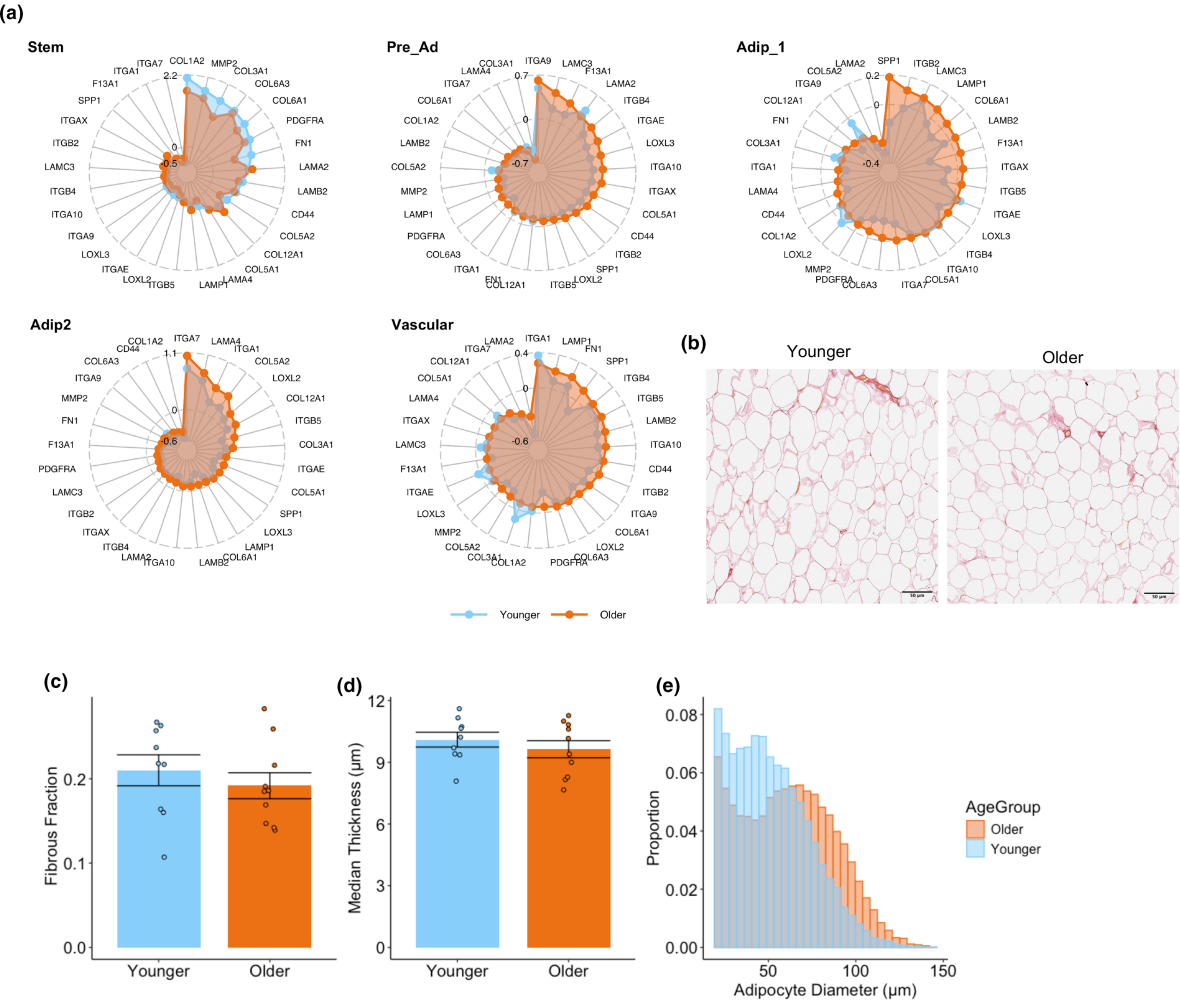
Fibrosis in aging WAT. Radar plots of differentially expressed extracellular remodeling and fibrosis genes in different cell types highlighting increased fibrosis in the older group in non‐stem cell populations (a). Representative picrosirius red staining in white adipose tissue in younger and older group (b). The fraction of adipose tissue with picrosirius red staining between younger and older group was not significantly different (c). The average median fibrosis thickness of each adipocyte between younger and older was not significantly different (d). Combined histogram of adipocyte size distribution normalized for total counts and split by age (e).

Top genes contributing to upregulated pathways are displayed in Figure 3c. Several DEGs upregulated in the Pre‐adipocytes, Adipocyte populations and Vascular populations in the Older Group were also related to ECM matrix remodeling and fibrosis (e.g., collagens [*COL1A2*, *COL4A2*, etc], integrins [*ITGB5*, *ITGA1*], laminins [*LAMA4*, *LAMA2*], fibronectin [*FN1*], etc). Collagen and ECM remodeling are an essential part of healthy adipose tissue growth (Johnston & Abbott, 2022); however, overexpression of collagens is associated with fibrotic tissue, obesity, and insulin resistance (Lackey et al., 2014; Lawler et al., 2016; Sun et al., 2014). We identified 29 differentially expressed genes related to ECM remodeling and fibrosis between older and younger groups in the different cell types and sought to determine how their expressions are differentially regulated between cell types (Figure 4a). While ECM molecules can be produced by adipocytes, the majority of collagens are produced by cells from the stromal vascular fraction such as mesenchymal stem cells and mast cells (DeBari & Abbott, 2020; Jones et al., 2020; Sun et al., 2023). There were no DEGs between older and younger groups for mast cells, but the older group did have a higher proportion of mast cells. Radar plots highlight that younger stem cells upregulate fibril‐forming collagens (*COL1A2*, *COL3A1*), basement filament forming collagens (*COL6A3*, *COL6A1*), matrix metallopeptidase (*MMP2*), fibronectin (*FN1*), and fibrotic marker (*PDGFRA*) (Figure 4a), which is anticipated with fibroblast‐like stem cells. Conversely, the older group display an upregulation of bead‐filament forming collagens (*COL6A1*, *COL6A3*), fibril‐forming collagens (*COL5A2*), fibril‐associated collagen (*COL12A1*), integrins that interact with ECM proteins (*ITGB2*, *ITGB4*, *ITGA7*, etc.), ECM glycoprotein osteopontin (OPN) (*SPP1*), laminin subunits (*LAMC3*, *LAMB2*, *LAMA2*, etc.) and lysyl oxidases (*LOXL2*, *LOXL3*) in pre‐adipocyte, adipocyte, and vascular cells (Figure 4a). Collagens V, VI, and XII are upregulated in fibrotic tissue and associated with impaired insulin sensitivity (Divoux et al., 2010; Kaartinen et al., 2022; Khan et al., 2009; Spencer et al., 2010), while lysyl oxidases can cross link collagens (Csiszar, 2001) and promote fibrosis (Halberg et al., 2009). Given that the transcriptional profile of older WAT suggested greater degrees of fibrosis, we next quantified fibrosis in WAT histological sections with picrosirius red staining (Figure 4b). There were, however, no significant differences in total levels of fibrosis measured across the whole WAT section or average median fibrosis thickness per adipocyte (Figure 4c,d).

### 2.5 Adipocyte size

Inflammation is associated with adipocyte hypertrophy. Differences in adipocyte size were determined by histology (Honecker et al., 2021) with an average of 1046 adipocytes measured per participant. The histogram plot of adipocyte diameter normalized to total counts shows a shift towards larger adipocytes with aging (Figure 4e). While mean adipocyte diameter (μm) was not statistically different between the older and younger groups (Figure S4A), the older group had a significantly greater proportion of adipocytes greater than 100 μm (hypertrophic adipocytes) (Trim et al., 2022) compared to younger (Figure S4B).

### 2.6 Macrophage content

**FIGURE 5:**
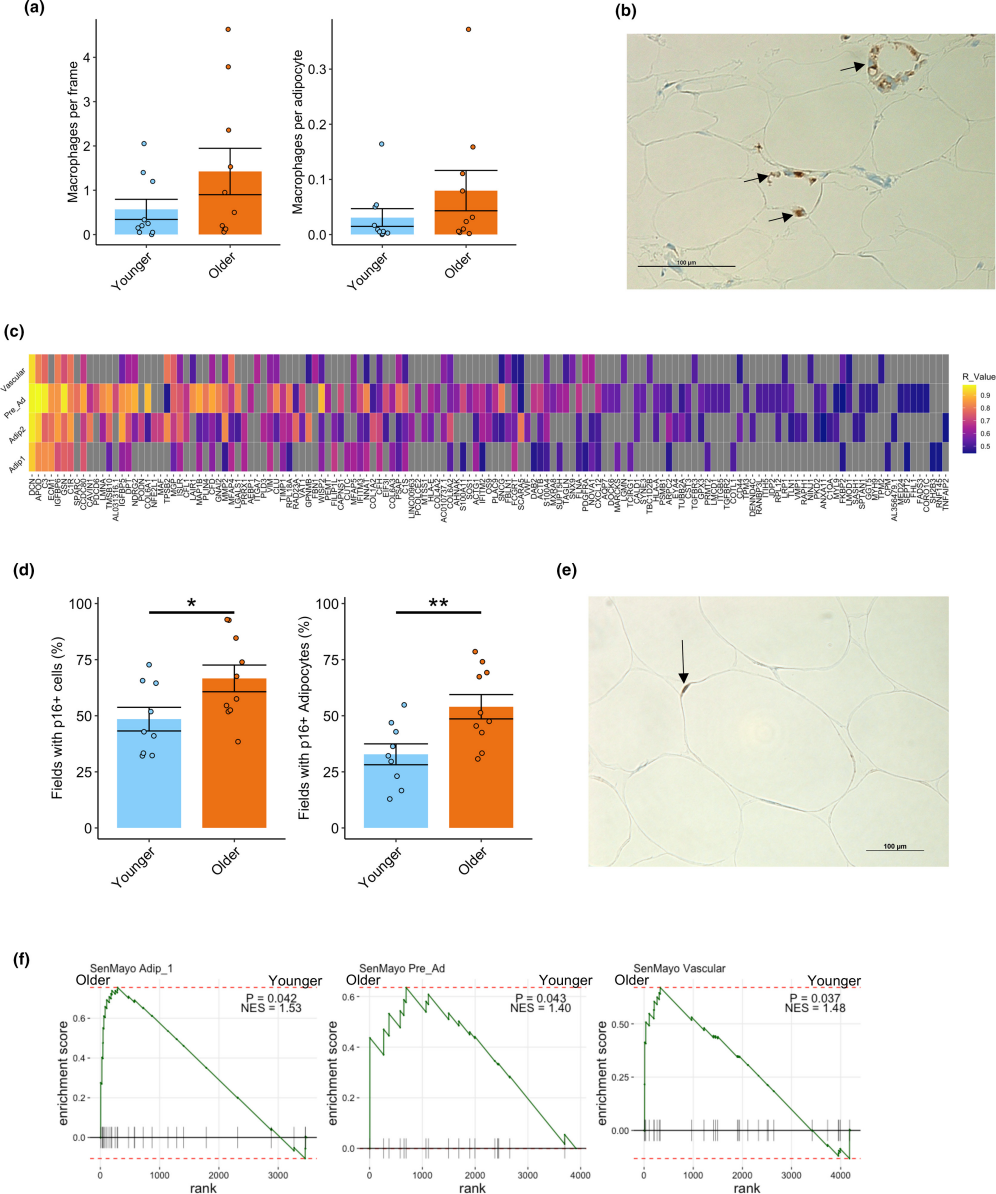
Macrophages and senescence in aging WAT. The number of macrophages per frame and per adipocytes stained for CD68 was not significantly different between the younger and older group (a). Representative image of macrophage crown‐like structure in the older group stained for CD68 (b). Heatmap of genes that positively correlated with *CXCL14* expression in cell types that show markers of inflammation and fibrosis in the older group (c) Tiles that are grey do not have significant positive correlation to *CXCL14* expression for that cell type. The percentage of 20X magnification fields that had cells and adipocytes with p16 staining was greater in older compared to younger participants (d). Representative image of p16 cell staining from an older individual with p16 in and adipocyte highlighted (e). GSEA plots of the SenMayo gene set showing greater enrichment in older compared to younger participants for Adip_1, Pre_Ad and Vascular cells (f). *p < 0.05; **p < 0.01. GSEA, gene set enrichment analysis.

Given our snRNA‐seq data show a greater number of LAM in the older group, we quantified macrophage content via immunohistochemistry using a pan‐macrophage marker CD68 (Weisberg et al., 2003). The numbers of macrophages quantified with CD68 per field of view and per adipocyte were not statistically significant between the older and younger groups (Figure 5a). Three participants had crown‐like structures of macrophages (Figure 5b), which form around dying or damaged adipocytes (Cinti et al., 2005). All of these individuals were males, and two of them were from the older group. Obesity is associated with macrophage infiltration (Weisberg et al., 2003). In agreement, we noted a significant and positive correlation between a marker of abdominal obesity, waist‐hip ratio (WHR), and the number of macrophages per adipocytes (Figure S5A). WHR was greater in the older group (Table 1), and this was driven specifically by older males (Figure S5B). We therefore reason that overall macrophage infiltration occurs in older males with higher WHR that can lead to crown‐like structures developing around damaged adipocytes.

Given the males in the older group had greater abdominal obesity (WHR), we sought to distinguish the effects of aging from the effects of greater WHR in WAT. We identified genes that were positively correlated with WHR in the four cell clusters that displayed inflammation (Adip_1, Adip_2, Pre_Ad, Vascular) (Table S6) and compared them to genes upregulated in the older group for these cell types (Figure S5C). While there was some overlap (1%–11%) between genes positively correlated with WHR and genes upregulated in the older group, there were more genes that were distinct. Therefore, aging is driving independent cell‐type transcriptional changes associated with inflammation and is also associated with greater abdominal adiposity in the male population, which has a distinct transcriptional profile.

### 2.7 CXCL14

*CXCL14* was one of the top DEGs in the older group in Pre_Ad, Adip_1 and Adip_2 cells and was also positively correlated with WHR in Adip_1, Adip_2, and Vascular cell types (Table S6). CXCL14 is notable for its chemotactic properties (Lu et al., 2016), but has recently been identified as a mediator of macrophage communication in brown adipose tissue (Cereijo et al., 2018), as well as a regulator of insulin‐mediated glucose uptake in 3 T3‐L1 adipocytes (Takahashi et al., 2007). We sought to identify which genes are co‐expressed with *CXCL14*. We ran correlation analyses between *CXCL14* expression with the differentially upregulated genes in the older group for the cell types that displayed inflammation (Adip_1, Adip_2, Pre_Ad, and Vascular). Genes that are positively correlated with *CXCL14* expression are highlighted in Figure 5c for each cell type. A number of these genes were linked to inflammation and fibrosis indicating that *CXCL14* may be a new biomarker of these processes in WAT. Furthermore, the Pre‐Ad population had the highest number of positively correlated genes to *CXCL14*, which may indicate the inflammatory/re‐modeling process is intensified in this particular cell type.

### 2.8 Senescence

Cellular senescence is caused by accumulation of DNA damage (Campisi & d'Adda di Fagagna, 2007) and other cellular stressors (Swanson et al., 2013; Tchkonia et al., 2013; Zhu et al., 2014) leading to cells that are resistant to regulated cell death (Wang, 1995) and secrete chemokines, cytokines, growth factors, and matrix metalloproteinases (Coppé et al., 2008) that can negatively impact the function of other cells in the microenvironment (Xu, Palmer, et al., 2015; Xu, Tchkonia, et al., 2015). Senescence typically occurs in proliferating cell types but has also been found in terminally differentiated cells (Farr et al., 2016; Jurk et al., 2012) including adipocytes (Justice et al., 2018; Li et al., 2021).

Given that the older group had a greater proportion of larger (>100 μm) adipocytes, upregulated inflammation and fibrosis markers, we reasoned that the older group would have increased levels of senescence, which may occur specifically in their adipocytes. We performed immunohistochemistry staining for the classic senescent marker p16. Abundance of p16+ cells was quantified as the percentage of (1) total fields analyzed containing p16+ cells (Justice et al., 2018) and (2) p16+ adipocytes. The older group had significantly more fields containing p16+ cells than the younger group (Figure 5c,d). The percentage of fields containing p16+ adipocytes was also greater among older individuals (Figure 5c,d), suggesting the increased levels of tissue senescence may be driven at least partially by adipocytes. Therefore, we show for the first time in human WAT that increased adipocyte senescence occurs concomitantly with increased adipocyte hypertrophy. p16 (CDKN2A) and p21 (CDKN1A) are canonical markers of senescence but are unreliable in detecting senescence at the transcriptional level due to very low expression. To measure senescence profiles in our snRNA‐Seq data set, we performed GSEA on older versus younger for each cell type using the recently published SenMayo gene set which identifies the transcriptional profile of senescent cells with a combination of 125 genes (Saul et al., 2022). Older Adip_1, Pre_Ad, and Vascular cells were enriched for SenMayo genes (Figure 5e), whereas other cell types showed no significant enrichment. Therefore, the increased p16+ adipocytes observed with immunohistochemistry may specifically be driven by Adip_1 population rather than Adip_2.

Due to senescence typically occurring in proliferating stem cells (Campisi & d'Adda di Fagagna, 2007), we also explored whether different stem cell subpopulations show more of a senescence profile and whether the proportions are altered with age. We subclustered our stem cells into 4 distinct clusters (Figure S6A) and identified them with known marker genes identified from previous publications (Figure S6B) (Hepler et al., 2018; Merrick et al., 2019; Sárvári et al., 2021; Schwalie et al., 2018). Stem_1 had an upregulation of *C3*, *GSN* and *COL6A2* and are likely more stem‐like (Cescon et al., 2023). Stem_2 had an upregulation of *F3* (CD142), which is marker of adipogenesis‐regulatory (Aregs) cells that are refractory to adipogenesis (Merrick et al., 2019; Schwalie et al., 2018), and aligned with FAP1 marker *FOXP2* from Sárvári et al. (2021). Stem_3 had an upregulation of lipid metabolism genes *CD36*, *FABP4 G0S2*, and *PNPLA2* indicating they are committing to the pre‐adipocytes lineage (Hepler et al., 2018; Sárvári et al., 2021; Schwalie et al., 2018). Stem_4 had upregulation of fibro‐inflammatory progenitor markers *CD9*, *FN1*, *FBN1*, and *DPP4* highlighted in previous research (Hepler et al., 2018; Merrick et al., 2019; Sárvári et al., 2021). Stem_1 and Stem_3 had a significant positive and negative enrichment of SenMayo genes, respectively (Figure S6C), whereas Stem_2 and Stem_4 showed no significant enrichment. Therefore, out of the stem‐like progenitor cells, Stem_1 had the greatest senescence profile. There were, however, no differences in proportions of any subpopulations of any stem cells between older and younger individuals indicating stem cell senescence in adipose tissue is not increased with age (Figure S6D).

## 3 DISCUSSION

We present a single nuclei atlas of aging human abdominal subcutaneous WAT in the largest prospective cohort study to date using cutting‐edge full‐length transcriptional profiling. We highlight that aging WAT is associated with inflammation in adipocyte, pre‐adipocyte, and vascular cell populations, LAM and mast cell infiltration, senescence in a specific adipocyte population, and adipocyte hypertrophy.

In models of severe obesity, it has been well characterized that WAT contains hypertrophic adipocytes linked to acute hypoxia and production of proinflammatory cytokines, which drives influxes of mast cells and macrophages to assist in cell death and removal (DeBari & Abbott, 2020; Sun et al., 2023). During this process, ineffective ECM remodeling can lead to WAT fibrosis, which can affect plasticity, and function of the tissue (Divoux et al., 2010). In this manuscript, we present a similar yet less exaggerated model that occurs in the context of aging. In agreement with previous findings (Trim et al., 2022), the older group had a shift towards larger adipocytes, which have previously been associated with hypoxia, oxidative stress, and inflammation (Haczeyni et al., 2018; Muir et al., 2016). While hypoxia was not identified transcriptionally in the older group in our dataset, there were other putative signals that aging WAT is under‐going remodeling. The vascular, adipocyte, and pre‐adipocyte populations had upregulated pathways related to inflammation in the older group (e.g., complement, interferon gamma response, apoptosis), which was paralleled by an elevation of LAM and mast cells.

Macrophages are the dominant leukocyte present in WAT (Russo & Lumeng, 2018). Adipocytes from obese mice produce chemokines and cytokines that activate monocyte differentiation into M1 or pro‐inflammatory macrophages (otherwise referred to as LAM) (Xu et al., 2003). Quantification of macrophage content and composition in aging WAT has been limited. Aging visceral adipose tissue (VAT) in mice shift towards a more pro‐inflammatory M1 phenotype (Lumeng et al., 2011). In humans, there has been previous evidence that macrophage content (quantified by CD68^+^ immunohistochemistry) increases and then declines with aging in subcutaneous WAT; however, this analysis was restricted to an age range of 18–45 years old and was in a cohort of Pima‐Indians only (Martinez et al., 2009). Recently, Trim et al. (2022) showed no differences in the proportion of CD206+ macrophages analyzed by FACS of the stromal vascular fraction between younger (20–35 years old) and older (60–85 years old) individuals; however, CD206 (*MRC1*) is considered an M2 anti‐inflammatory or resident macrophage marker. Thus, to observe macrophage content changes with aging, it is critical to profile and distinguish different macrophage sub‐types and phenotypes. By using snRNA‐Seq on whole WAT, we confirmed an increase in LAMs (M1) that typically exhibit a more pro‐inflammatory phenotype. Recently, Hildreth et al. (2021) used FACS to sort deep subcutaneous adipose tissue derived from abdominoplasty surgery into CD45+ and CD45‐ cells and performed scRNA‐Seq on both fractions. In this analysis they were further able to resolve macrophages into 3 distinct populations characterized as perivascular macrophages (similar to resident macrophages), lipid‐associated macrophages, and an uncharacterized inflammatory macrophage (Hildreth et al., 2021). Without any prior FACS enrichment and due to using whole tissue including adipocytes, we were not able to resolve the macrophage populations to the same level. Unlike other sc/snRNA‐Seq datasets (Emont et al., 2022; Hildreth et al., 2021), we were unable to detect NK/T cells. While the full‐length snRNA‐Seq ICELL8 platform has enhanced gene detection capabilities, it is restricted by the quantity of nuclei that can be profiled for one sample (1100–1600 nuclei), which restricts resolving certain immune cell profiles due to their low abundance. Future research should FACS purify immune cells from older and younger groups prior to performing scRNA‐Seq to comprehensively interrogate immune cell populations with aging.

Mast cells can contribute to low‐grade inflammation by secreting inflammatory mediators and attracting other immune cells (Metz et al., 2007; Rao & Brown, 2008). In WAT mast cells are associated with macrophage accumulation, endothelial cell inflammation, insulin resistance, and fibrosis (Divoux et al., 2012; Gurung et al., 2019). For the first time, we highlight that mast cell composition increases with aging human WAT. Fibrosis staining is elevated in WAT of aged mice (Donato et al., 2014). The transcriptional profiles in the current study suggested that pre‐adipocyte, adipocytes, and vascular cells showed early stages of a fibrotic phenotype in the older group; however, this was not confirmed histologically with picrosirius red staining in the WAT of our cohorts. Therefore, while aging WAT is undergoing inflammation and remodeling, it has not yet reached the detrimental stage of overt fibrosis. A significant and critical aspect of our study populations is that the older group had comparable levels of plasma FFA, plasma insulin, and WAT insulin sensitivity, compared to the younger group, suggesting WAT function was not compromised in these individuals.

There is recent interest in mapping senescent cells for future senolytic or other intervention‐based approaches (SenNet Consortium, 2022). Prolonged caloric restriction (12 and 24 months) was recently shown to decrease the senescence transcriptional profile in human WAT (Aversa et al., 2023); however, these bulk sequencing approaches do not determine which cell types are most responsive to change. After confirmation of increased p16 staining in histological sections in the older group that was driven by p16+ adipocytes, we sought to identify which adipocytes are responsible for the increased senescence. Using the SenMayo GSEA, we identified that vascular cells, pre‐adipocytes, and Adip_1 (anti‐oxidative) all displayed enrichment in senescence in the older group, while Adip_2 (insulin‐responsive) did not show senescence enrichment. Therefore, these specific populations of adipocytes should be targeted in future senolytic‐driven research.

It is well established that aging is associated with a redistribution of adipose to centrally located abdominal depots (Palmer & Kirkland, 2016). In support, WHR was elevated in the older group, and this was driven by the older males. However, our results dissociate the effect of WHR on the transcriptional profile of cells from that observed with aging. Therefore, while both abdominal obesity and aging can have complementary negative effects on WAT, they likely originate from diverse transcriptional pathways.

We highlight *CXCL14* as marker of aging particularly in individuals with greater abdominal obesity (WHR) and discovered that *CXCL14* strongly correlated with genes associated with fibrosis and ECM remodeling. The role of CXCL14 in WAT has been confounded by a lack of consensus identifying and validating its receptor; however, it was recently proposed that CXCL14 synergizes with low concentrations of CXCL13 and CCL19/CCL21 during in vitro chemotaxis with immune cells expressing receptors CXCR5 and CCR7 (Kouzeli et al., 2020). CXCL14 is known to recruit macrophages (Cereijo et al., 2018); however, its expression in WAT and circulating concentrations is reduced with metabolic abnormalities such as obesity, PCOS, and T2D (Cereijo et al., 2021; García‐Beltran et al., 2020). We noted the Pre‐Ad population had the highest number of positively correlated genes to *CXCL14* indicating the inflammatory/re‐modeling process is intensified in this cell type. *SPARC* was among one of the highest correlating genes to *CXCL14* in pre‐adipocytes. SPARC has also been shown to activate macrophages via JNK signaling and can be downregulated with caloric restriction aimed at improving longevity (Ryu et al., 2023). Given these new findings, future research should focus on the role of *CXCL14* in mediating macrophage activation in aging WAT and if this is specific to the individuals with greater WHR.

We identified two distinct adipocyte populations that aligned with adipocyte populations identified with spatial transcriptomics of WAT (Bäckdahl et al., 2021). This finding is novel when compared with snRNA‐Seq of WAT performed using the 10X platform at a lower sequencing depth (Emont et al., 2022; Sárvári et al., 2021), which have identified one main adipocyte population prior to higher resolution sub‐clustering. We characterized these two adipocyte populations based on their transcriptional profiles without sub‐clustering, and further investigation is required to validate their representative biological processes.

In conclusion, aging WAT is associated with low‐grade inflammation in pre‐adipocyte, adipocyte, and vascular cell populations, enhanced senescence in specific adipocyte populations, paralleled by an influx of mast cells and LAMs. We highlight CXCL14 as a marker of aging and abdominal obesity that is positively correlated with genes associated with fibrosis and ECM remodeling. We confirm these transcriptional and phenotypical changes occur without overt fibrosis and in older individuals that have WAT insulin sensitivity comparable to younger individuals.

## 4 Experimental model and subject details

Younger (≤30 years old) and older (≥65 years old) individuals were recruited to the TRI at AdventHealth to participate in the study. All participants were free from metabolic and infectious disease, were not taking medication related to diabetes or inflammation, and had not had major surgery within the last 4 weeks. Five participants were taking one or more medications to treat the following conditions; hypertension, hyperlipidemia, urinary retention, thyroid, anxiety, and osteoporosis. All participants were weight‐stable for at least 3 months prior to the assessments and adipose biopsy. The study was approved by AdventHealth Institutional Review Board and carried out in accordance with the Declaration of Helsinki. Participants provided written informed consent to partake in the study. All samples were taken following an overnight fast.

Abdominal subcutaneous WAT biopsies were performed following an overnight fast using the tumescent lidocaine approach with a Mercedes aspiration cannula (Divoux et al., 2018). Following removal of excess blood and connective tissue, the sample was cleaned with PBS. A portion (∼100 mg) was immediately snap frozen for subsequent nuclei isolation for snRNA‐seq. A portion (∼20 mg) was fixed in 10% formalin for 24 h and stored in 70% EtOH for subsequent histological analyses.

### 4.1 Nuclei isolation from whole white adipose tissue

Nuclei were isolated from frozen WAT as previously detailed (Whytock et al., 2023). WAT was pulverized under linked nitrogen before being homogenized in 2 mL of homogenization buffer (5 mM MgCl_2_, 25 mM Tris Buffer pH 8.0, 25 mM KCL, 250 mM sucrose, 1 μm DDT, 1× protease inhibitor, 0.2 U/μL SUPERase in RNase Inhibitor [Thermofisher Scientific] in nuclease‐free water) with a glasscol homogenizer. Following addition of Triton‐X100 (0.1% v/v), the homogenate was incubated on ice with regular vortexing. Samples were then filtered through a 100 and 40 μm strainer (BD Falcon), centrifuged at 2700 *g* for 10 min at 4°C, resuspended in homogenization buffer, and recentrifuged again at 2700 *g* for 10 min at 4°C. The pellet was then re‐suspended in 1 mL nuclei isolation medium (5 mM MgCl_2_, 25 mM Tris Buffer pH 8.0, 25 mM KCL, 1 mM EDTA, 0.2 U/μL Ribolock RNAase inhibitor, 1% BSA in nuclease‐free water) before being centrifuged at 2700 *g* for 10 min at 4°C. The sample was re‐suspended in 500 μL nuclei isolation medium before being filtered 10× with a 25‐g syringe. Nuclei were stained with Hoechst 33342 (ReadyProbes Cell Viability Imaging Kit, Thermofisher Scientific) and counted with a countess II automated cell counter (Thermofisher Scientific).

### 4.2 Single‐nuclei RNA‐Seq

Single‐nuclei suspension (40 K/mL) was aliquoted into 8 wells of a 384‐well source plate (Takara Bio USA, San Jose, CA) and dispensed using an iCELL8 MultiSample NanoDispenser (Takara Bio USA) onto an iCELL8® 350v Chip (Takara Bio USA). Following dispense, the chip nanowells were imaged using the iCELL8 Imaging Station to identify nanowells containing a single nucleus, with only these nanowells being subjected for downstream dispenses. After imaging, the chip was subjected to freeze–thaw to lyse the nuclei, followed by a 3‐min incubation at 72°C to denature the RNA. Selected nanowells were subjected to first‐strand cDNA synthesis initiated by oligo dT primer (SMART‐Seq iCELL8 CDS), followed by template switching with template switching oligo (SMART‐Seq iCELL8 oligonucleotide) for 2nd‐strand cDNA synthesis, before unbiased amplification of full‐length cDNA. Tagment DNA enzyme 1 (TDE1, Illumina, San Diego, CA) was used to tagment full‐length cDNA before amplification with forward (i5) and reverse (i7) indexing primers. Each single nucleus was indexed by a unique combination of 1 of 72 forward and 1 of 72 reverse indexing primers allowing for downstream identification. Collected cDNA was purified twice using a 1:1 proportion of AMPure XP beads (Beckman Coulter, Brea, CA). cDNA was further amplified according to manufacturer's instructions and purified again at a 1:1 proportion of AMPure XP beads. The resultant cDNA library was assessed for concentration by fluorometer (Qubit, Thermofisher Scientific) and quality by electrophoresis (Agilent Bioanalyzer high‐sensitivity DNA chips). Libraries were sequenced with Illumina HiSeq 4000 at an average sequencing depth of 270 M per library. This equated to an average 118,568 barcoded reads per nuclei.

#### 4.2.1 Bioinformatic analyses

Demultiplexing, mapping, alignment, and counting of the single nuclei RNA‐Seq libraries were performed using CogentAP™ Analysis Pipeline (Takara Bio, USA). GRCh38 was used as the genome reference and included 58,735 genes. Cell and gene filtering was performed in R package scran and scuttle (Lun et al., 2016). Nuclei were initially filtered if they had; <500 genes, >20,000 genes, <10,000 counts, >30% mitochondrial reads or if cell complexity was <0.65. Following initial filtering, outliers were removed based on three median absolute deviations of log total counts. To remove low expressed genes from clustering analyses, genes with an average count less than 0.1 were removed. Data was normalized in Seurat with SCTransform (Hafemeister & Satija, 2019; Hao et al., 2021).

Each sample was initially clustered separately using 5000 highly variable protein‐coding genes to detect sample‐dependent cell‐type clusters. Data was adjusted for ambient RNA with decontx (Yang et al., 2020) with cell cluster labels used as the Z parameter. When data sets were merged and clustered, there were moderate batch effects resulting in two cell clusters being derived from only one sample (Figure S1A). We therefore compared multiple integration methods; STACAS, fastMNN, Seurat (RPCA), and harmony (Andreatta & Carmona, 2021; Hafemeister & Satija, 2019; Hao et al., 2021; Korsunsky et al., 2019; Zhang et al., 2019) on their ability to integrate samples while retaining biological variability (Figure S1). For each integration 5000 highly variable protein‐coding genes were used, and where applicable, the number of PCA dimensions used for integration was 1:20 and the number of PCA dimensions used for Uniform Maniform Approximation and Porjection for Dimension Reduction (UMAP) projection and clustering was (1:10). Adjusted rand index (ARI) (Hubert & Arabie, 1985) was calculated using the *adj.rand.index* function in *pdfCluster* v1.04. by providing a list of participant IDs and identified cell clusters. In this instance, an ARI of 1 would indicate samples have clustered based on participant rather than cell type. Whereas an ARI of 0 would indicate random clustering of samples among the different cell types and indicate good batch correction. Average Local Inverse Simpson's index (LISI) scores were estimated using the *compute_lisi* function in *lisi* v1.0 (Korsunsky et al., 2019) by providing a list of the UMAP embeddings along with the participant ID and cell clusters to estimate how well‐mixed donors are across the cell clusters. As there were 20 samples, a score closer to 20 would indicate the cells are entirely mixed among participants, whereas 0 would indicate the cells are not mixed well. Following integration both STACAS and fastMNN (Figure S1B,C) still displayed cell clusters derived from individual samples and were therefore not suitable. Harmony in comparison to RPCA provided the highest LISI score and lowest ARI score indicating the samples were well integrated (Figure S1D,E). LISI scores were not greater than 10 indicating biological variability still exists within the integrated data set. We further optimized the clustering with harmony through number of PCAs (1:20) used for UMAP projection and clustering to derive more distinct cell clusters. This optimization preserved the ARI and LISI score (Figure S1F) and was used for further downstream analyses.

Differences in cell type proportions between groups were analyzed with the *propeller* function in *speckle* (Phipson et al., 2022). Differential gene expression analysis for cell clusters was performed using a Wilcoxon rank sum test with Seurat's “FindMarkers” function with a False Discovery rate (FDR) cutoff of <0.05, log_2_ FC > 0.25 or <−0.25 and expressed in >25% of nuclei in that cluster. Differential gene expression analysis between the older and younger group was performed using a Wilcoxon rank sum test with Seurat's “FindMarkers” function with a FDR cutoff of <0.05, log_2_ FC > 0.1 or <−0.1 and expressed in >10% of nuclei in that cluster. A hypergeometric test was used to assess over‐representation of upregulated genes (log_2_ FC < 0.25) in the older and younger group for each cell type using R package HypeR (Federico & Monti, 2020) querying datasets of Reactome and Hallmark (Gillespie et al., 2022; Liberzon et al., 2015). Significance was set at an FDR of 0.05. Genes detected in each cluster were used as a background reference. We ran GSEA (Subramanian et al., 2005) on SenMayo genes (Saul et al., 2022) using the R package FGSEA (Korotkevich et al., 2021). Gene ranking was performed by multiplying −log_10_ (*p* value) by the average log FC when comparing older and younger groups for each cell type.

### 4.3 Adipose tissue histology

White adipose tissue was fixed in 10% formalin, rinsed with PBS, stored in 70% EtOH, embedded in paraffin within 30 days, and sectioned at 5 μm. Slides were stained with picrosirius red, and images were captured with an Aperio AT2 scanner (Leica Biosytems, Germany). Immunohistochemical detection of CD68 (Atlas Antibodies, AMAb90873) for macrophages and p16 (Enzo Life Science, ENZ‐ABS664) was conducted with the avidin‐biotin peroxidase method. Images were captured using a Nikon Eclipse Ti microscope (Nikon Technologies, California) at 20× magnification.

Adipocyte size and fibrosity were quantified using FIJI software with the Adipose Tissue Analysis Toolkit (Robino et al., 2018) on 9 younger and 10 older samples. A region of interest was drawn within the section to exclude any folded tissues or artifacts from the analysis. Fibrosis fraction was determined as the fraction of the image stained darker then the threshold. Median pericellular fibrosis thickness and area were measured for each adipocyte. Adipocytes that were below 200 and above 16,000 μm^2^ were removed as they typically represent artefacts from processing (Honecker et al., 2021). Adipocyte area was converted to diameter.

Abundance of p16+ cells was quantified as the percentage of total fields (20× magnification) analyzed containing p16+ cells (Justice et al., 2018) and p16+ adipocytes over an average of 26 frames per participant. Head‐and‐neck cancer tissue was used as a positive control for the presence of p16. The presence of CD68‐positive cells was counted per frame with an average of 18 frames per participant and adjusted for the number of adipocytes per frame for each participant. Presence of crown‐like structures for each frame was also noted. Tonsil tissue was used as a positive control for the presence of CD68.

### 4.4 Blood metabolites

Fasting blood samples were collected for measurements of comprehensive metabolic panel, HbA1c (%), insulin, FFA, and CRP and analyzed in the clinical chemistry laboratory at AdventHealth using standard assays. Adipose tissue insulin resistance index (ADIPO‐IR) was calculated by multiplying plasma FFA (mmol/L) by serum insulin (pmol/L) (Groop et al., 1989; Søndergaard et al., 2017).

### 4.5 Statistical testing

For comparisons of clinical and phenotypical data between the older and younger groups, an unpaired *t*‐test was used to detect differences in normally distributed data, and Mann–Whitney *U* test was used for non‐normally distributed data. A two‐way ANOVA was used when comparing variables for age and sex with post‐hoc Tukey HSD.

## AUTHOR CONTRIBUTIONS

K.L.W. – Conceptualization, Methodology, Investigation, Formal analysis, Writing – Original Draft, Visualization. A.D. – Conceptualization, Methodology, Investigation, Writing – Review & Editing. Y.S. – Formal analysis, Investigation, Visualization, Writing – Review & Editing. M.F.P. – Methodology, Investigation, Writing – Review & Editing. G.Y. – Formal analysis, Visualization, Writing – Review & Editing. C.A.J. – Formal analysis, Writing – Review & Editing. J.J.R. – Methodology. A.P. – Methodology. O.V. – Methodology, Investigation, Writing – Review & Editing. S.R.S. – Conceptualization, Supervision, Funding Acquisition, Writing – Review & Editing. M.W. – Conceptualization, Methodology, Supervision, Writing – Review & Editing. L.M.S. – Conceptualization, Methodology, Supervision, Funding Acquisition, Writing – Review & Editing.

## FUNDING INFORMATION

The study was partially funded by James Vath, PhD and Huseyin Mehmet, PhD and was supported by an administrative supplement to grant #U01 AR071133 and by grant R01AG066474. Figure 1a and graphical abstract was generated with Biorender.com.

## CONFLICT OF INTEREST STATEMENT

The authors declare no conflict of interest.

## Supporting information


Figures S1–S6.

Tables S1–S6.

## Data Availability

The snRNA‐Seq data generated during this study have been deposited under GSE235529. Supplementary Tables can be accessed here https://figshare.com/s/949c28ec770f03954a29. Code Availability: Scripts used to process and analyze data in this paper have been deposited to GitHub. https://github.com/KWhytock13/aging‐wat/.
