# Supplementary material for: Aging human abdominal subcutaneous white adipose tissue at single cell resolution
Source: Aging Cell. 2024 Aug 14;23(11):e14287. doi: 10.1111/acel.14287 (PMC11561672; doi:10.1111/acel.14287)
Supplement: Supplementary file 1 — Figures S1–S6. [file ACEL-23-e14287-s002.pdf]

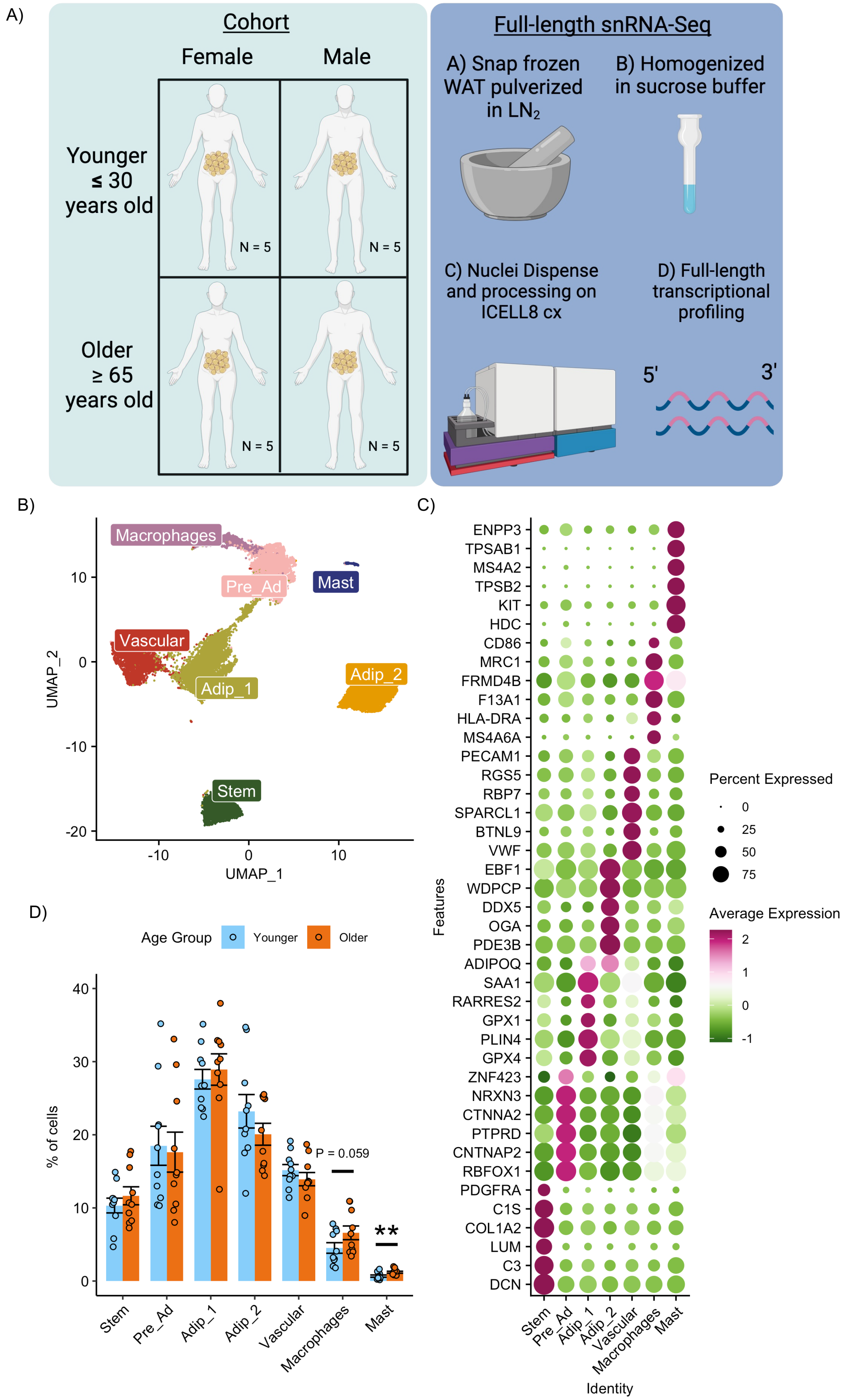
A)
Cohort
Female
Male
Younger
≤ 30
years old
Older
≥ 65
years old
N = 5
N = 5
N = 5
N = 5
Full-length snRNA-Seq
A) Snap frozen WAT pulverized in LN2
B) Homogenized in sucrose buffer
C) Nuclei Dispense and processing on ICELL8 cx
D) Full-length transcriptional profiling
5'
3'
B)
Macrophages
Pre_Ad
Mast
Vascular
Adip_1
Adip_2
Stem
UMAP_2
UMAP_1
C)
ENPP3
TPSAB1
MS4A2
TPSB2
KIT
HDC
CD86
MRC1
FRMD4B
F13A1
HLA-DRA
MS4A6A
PECAM1
RGS5
RBP7
SPARCL1
BTNL9
VWF
EBF1
WDPCP
DDX5
OGA
PDE3B
ADIPOQ
SAA1
RARRES2
GPX1
PLIN4
GPX4
ZNF423
NRXN3
CTNNA2
PTPRD
CNTNAP2
RBFOX1
PDGFRA
C1S
COL1A2
LUM
C3
DCN
Features
Identity
Percent Expressed
Average Expression
D)
Age Group
Younger
Older
% of cells
P = 0.059
**
Stem
Pre_Ad
Adip_1
Adip_2
Vascular
Macrophages
Mast

**Figure 1**

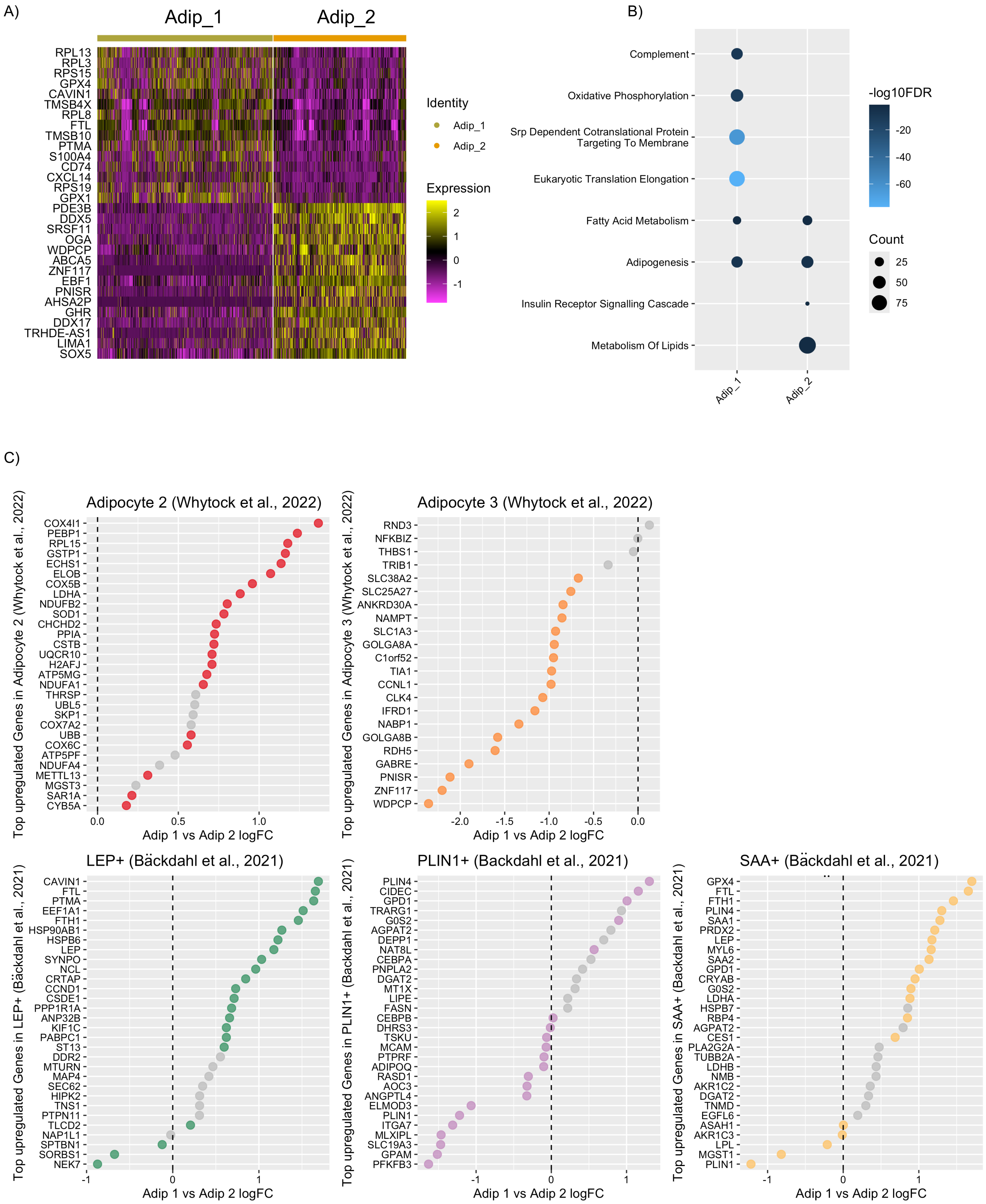
A)
Adip_1
Adip_2
RPL13
RPL3
RPS15
GPX4
CAVIN1
TMSB4X
RPL8
FTL
TMSB10
PTMA
S100A4
CD74
CXCL14
RPS19
GPX1
PDE3B
DDX5
SRSF11
OGA
WDPCP
ABCA5
ZNF117
EBF1
PNISR
AHSA2P
GHR
DDX17
TRHDE-AS1
LIMA1
SOX5
Identity
Adip_1
Adip_2
Expression
B)
Complement
Oxidative Phosphorylation
Srp Dependent Cotranslational Protein Targeting To Membrane
Eukaryotic Translation Elongation
Fatty Acid Metabolism
Adipogenesis
Insulin Receptor Signalling Cascade
Metabolism Of Lipids
-log10FDR
Count
C)
Adipocyte 2 (Whytock et al., 2022)
Top upregulated Genes in Adipocyte 2 (Whytock et al., 2022)
Adip 1 vs Adip 2 logFC
Adipocyte 3 (Whytock et al., 2022)
Top upregulated Genes in Adipocyte 3 (Whytock et al., 2022)
LEP+ (Bäckdahl et al., 2021)
Top upregulated Genes in LEP+ (Bäckdahl et al., 2021)
PLIN1+ (Backdahl et al., 2021)
Top upregulated Genes in PLIN1+ (Backdahl et al., 2021)
SAA+ (Bäckdahl et al., 2021)
Top upregulated Genes in SAA+ (Bäckdahl et al., 2021)

**Figure 2**

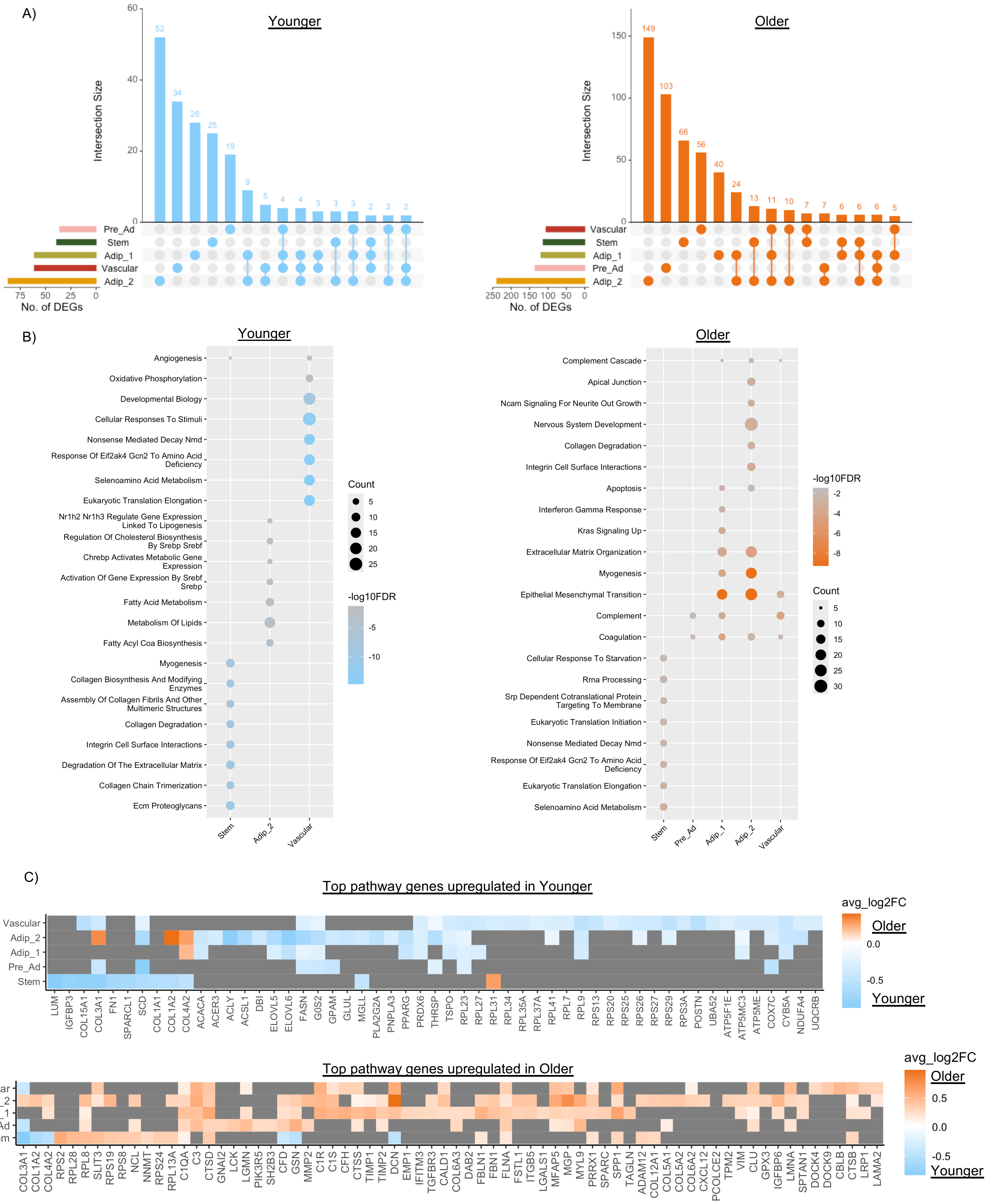
A)
Younger
Older
Intersection Size
No. of DEGs
Pre_Ad
Stem
Adip_1
Vascular
Adip_2
B)
Younger
Older
Count
-log10FDR
Angiogenesis
Oxidative Phosphorylation
Developmental Biology
Cellular Responses To Stimuli
Nonsense Mediated Decay Nmd
Response Of Eif2ak4 Gcn2 To Amino Acid Deficiency
Selenoamino Acid Metabolism
Eukaryotic Translation Elongation
Nr1h2 Nr1h3 Regulate Gene Expression Linked To Lipogenesis
Regulation Of Cholesterol Biosynthesis By Srebp Srebf
Chrebp Activates Metabolic Gene Expression
Activation Of Gene Expression By Srebf Srebp
Fatty Acid Metabolism
Metabolism Of Lipids
Fatty Acyl Coa Biosynthesis
Myogenesis
Collagen Biosynthesis And Modifying Enzymes
Assembly Of Collagen Fibrils And Other Multimeric Structures
Collagen Degradation
Integrin Cell Surface Interactions
Degradation Of The Extracellular Matrix
Collagen Chain Trimerization
Ecm Proteoglycans
Complement Cascade
Apical Junction
Ncam Signaling For Neurite Out Growth
Nervous System Development
Apoptosis
Interferon Gamma Response
Kras Signaling Up
Extracellular Matrix Organization
Epithelial Mesenchymal Transition
Complement
Coagulation
Cellular Response To Starvation
Rrna Processing
Srp Dependent Cotranslational Protein Targeting To Membrane
Eukaryotic Translation Initiation
C)
Top pathway genes upregulated in Younger
Top pathway genes upregulated in Older
avg_log2FC
Older
Younger

**Figure 3**

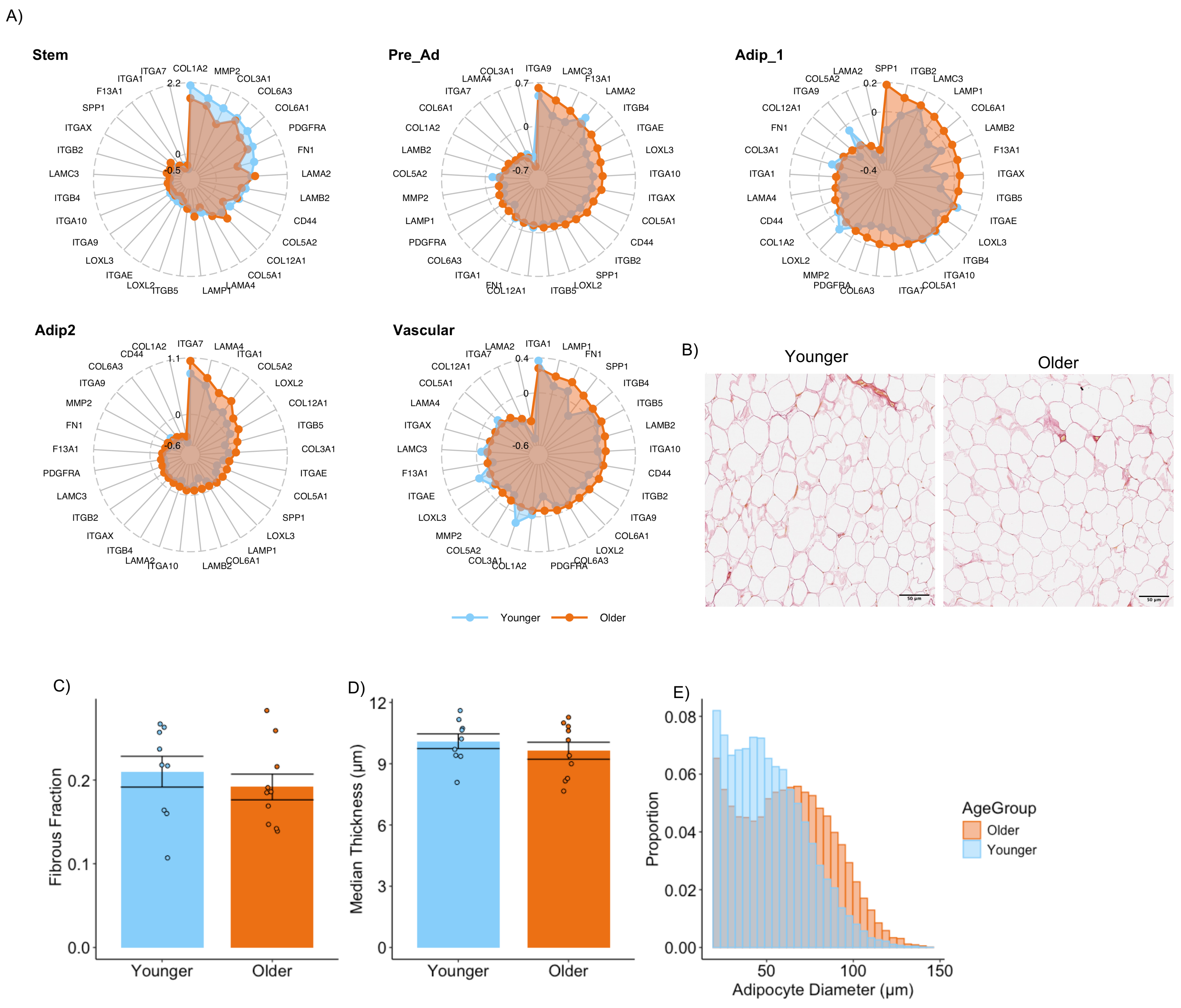

A)
Stem
Pre_Ad
Adip_1
Adip2
Vascular
Younger
Older
B)
Younger
Older
C)
Fibrous Fraction
Younger
Older
D)
Median Thickness (μm)
E)
Proportion
Adipocyte Diameter (μm)
AgeGroup
Older
Younger


**Figure 4**

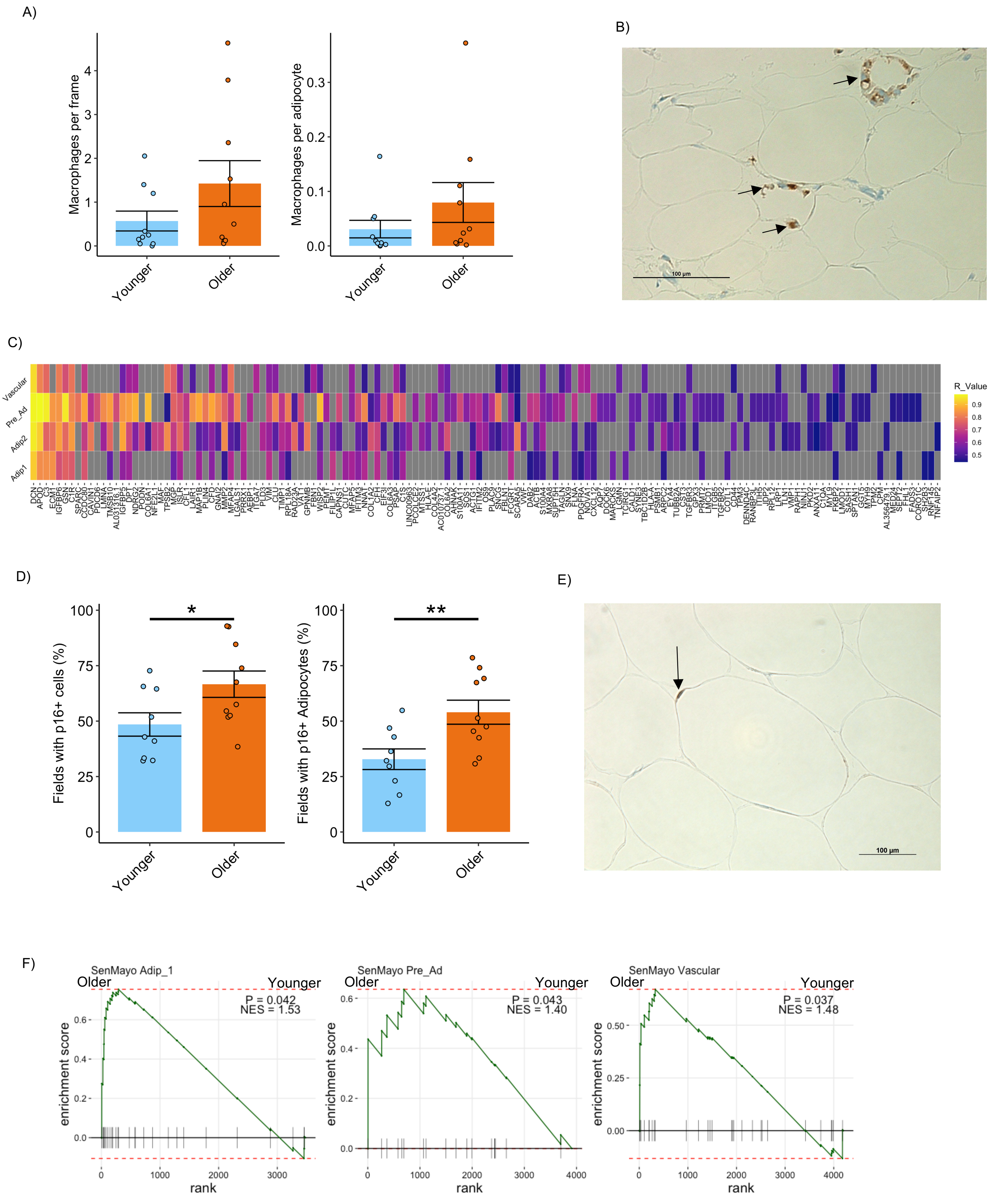

A)
Macrophages per frame
Macrophages per adipocyte
Younger
Older
B)
100 µm
C)
Vascular
Pre_Ad
Adip2
Adip1
R_Value
D)
Fields with p16+ cells (%)
Fields with p16+ Adipocytes (%)
*
**
Younger
Older
E)
100 µm
F)
SenMayo Adip_1
SenMayo Pre_Ad
SenMayo Vascular
Older
Younger
P = 0.042
NES = 1.53
P = 0.043
NES = 1.40
P = 0.037
NES = 1.48
enrichment score
rank


**Figure 5.**

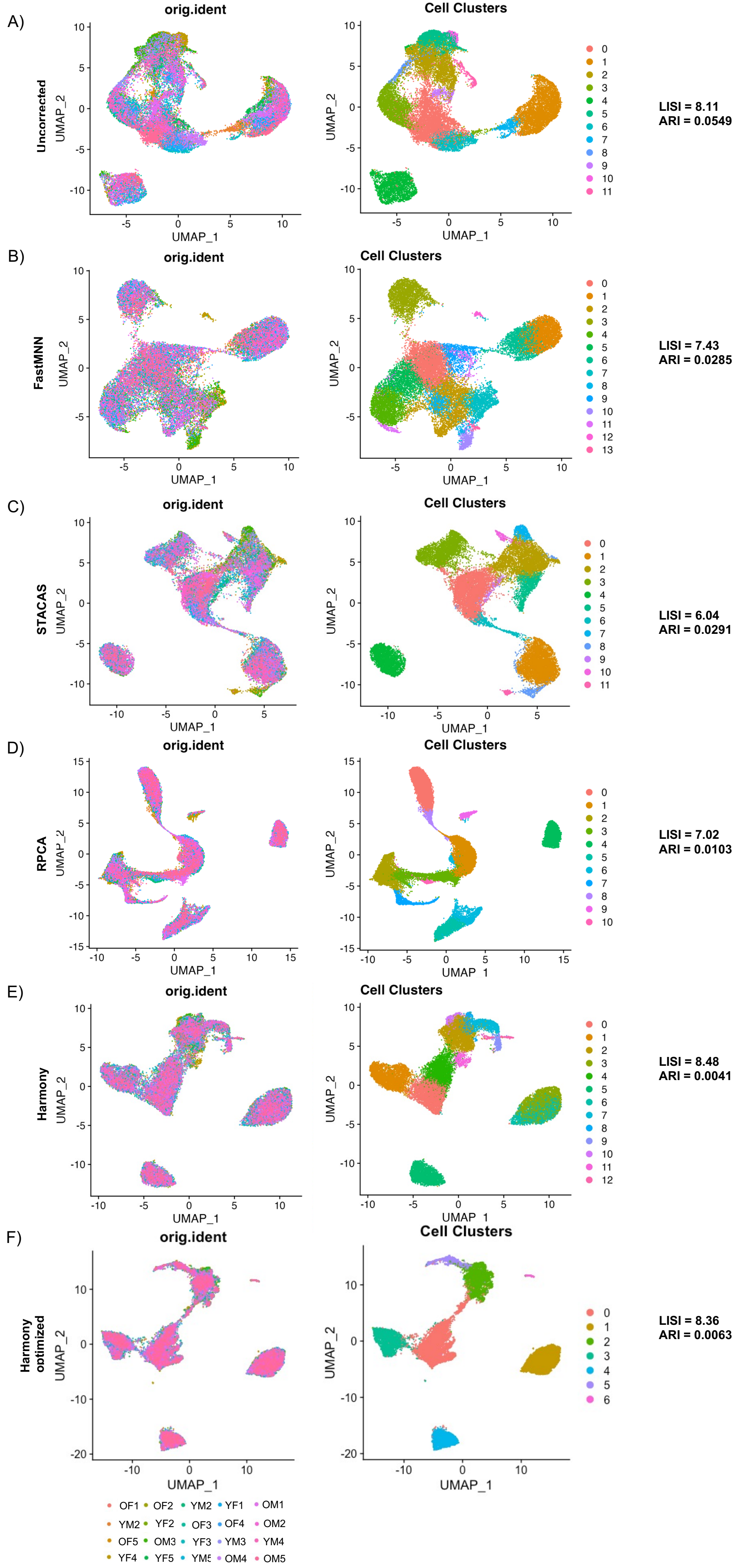


**Supplementary Figure 1**

A)

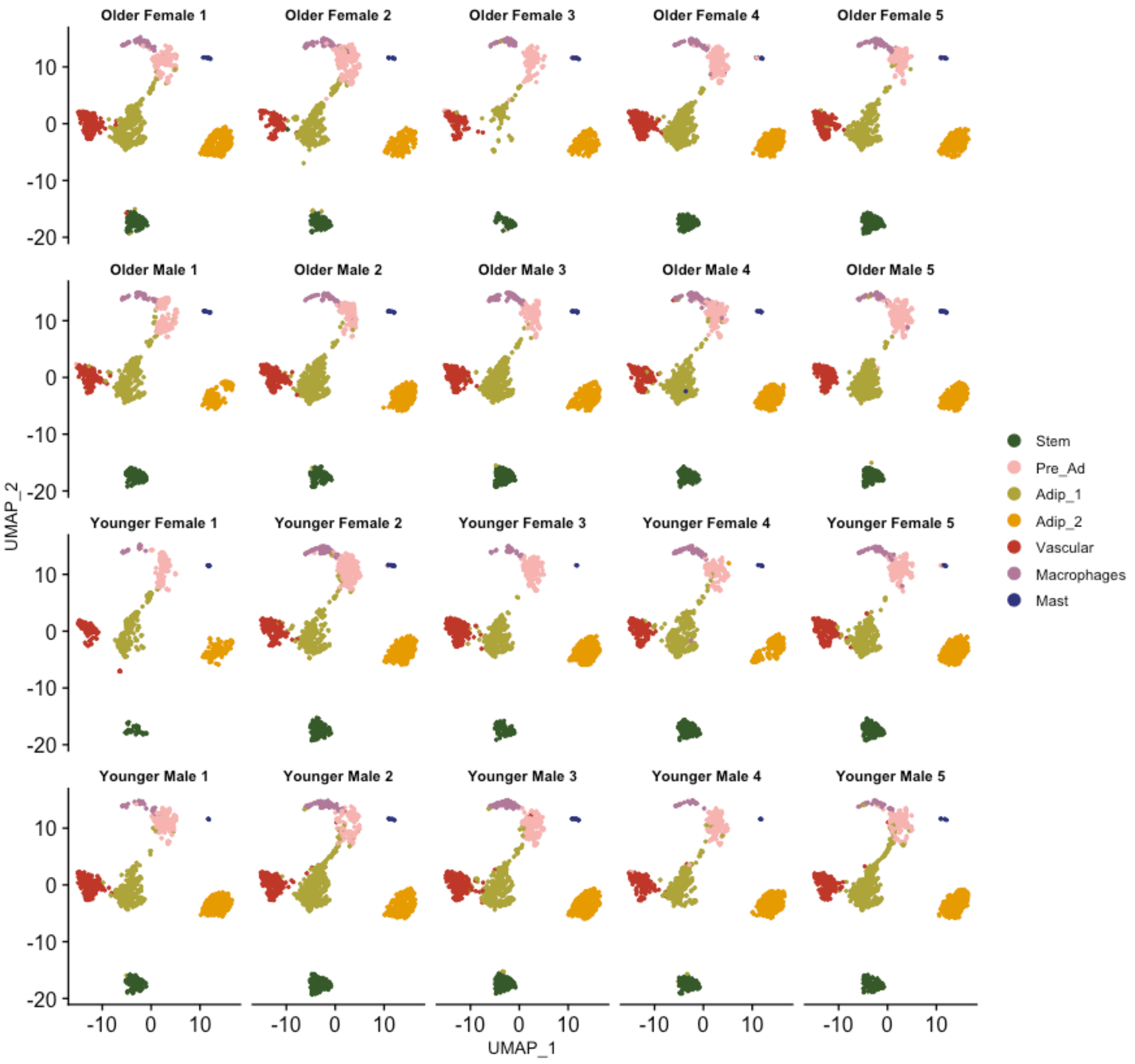


B)

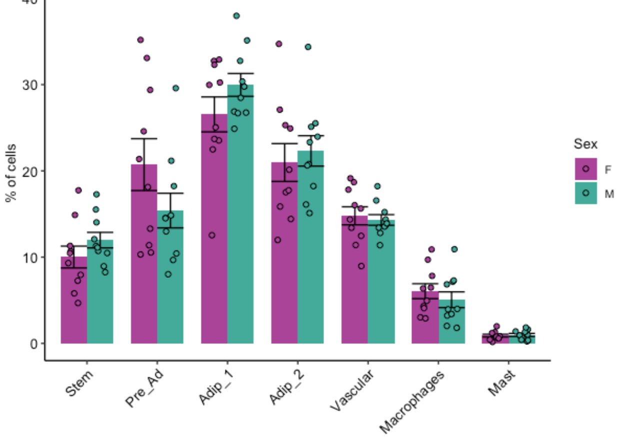


**Supplementary Figure 2**

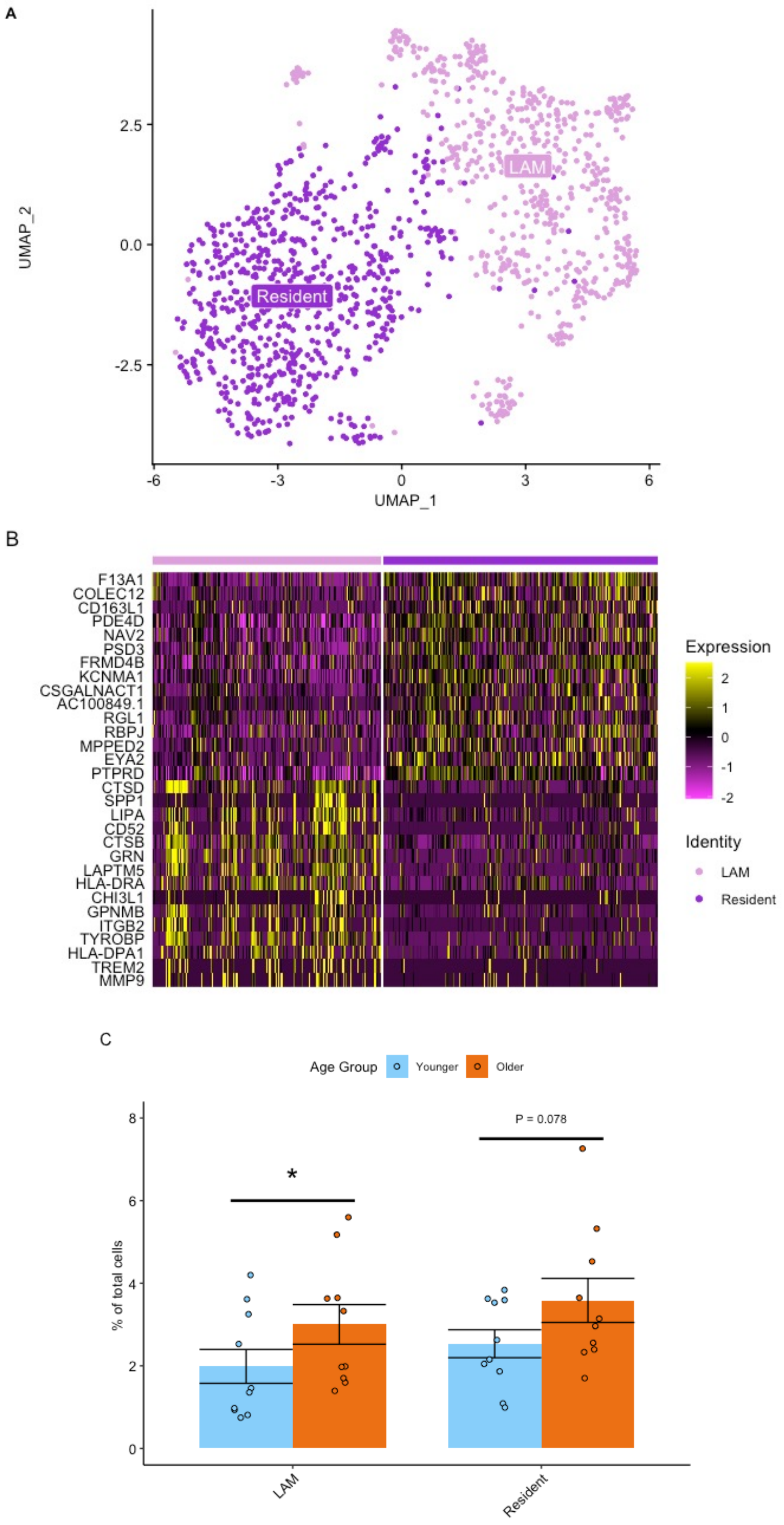


**Supplementary Figure 3**

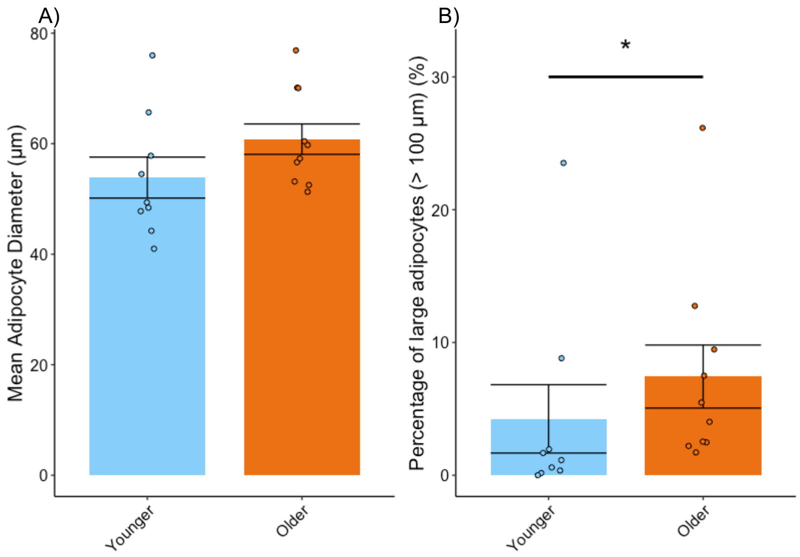


**Supplementary Figure 4**

A)

B)

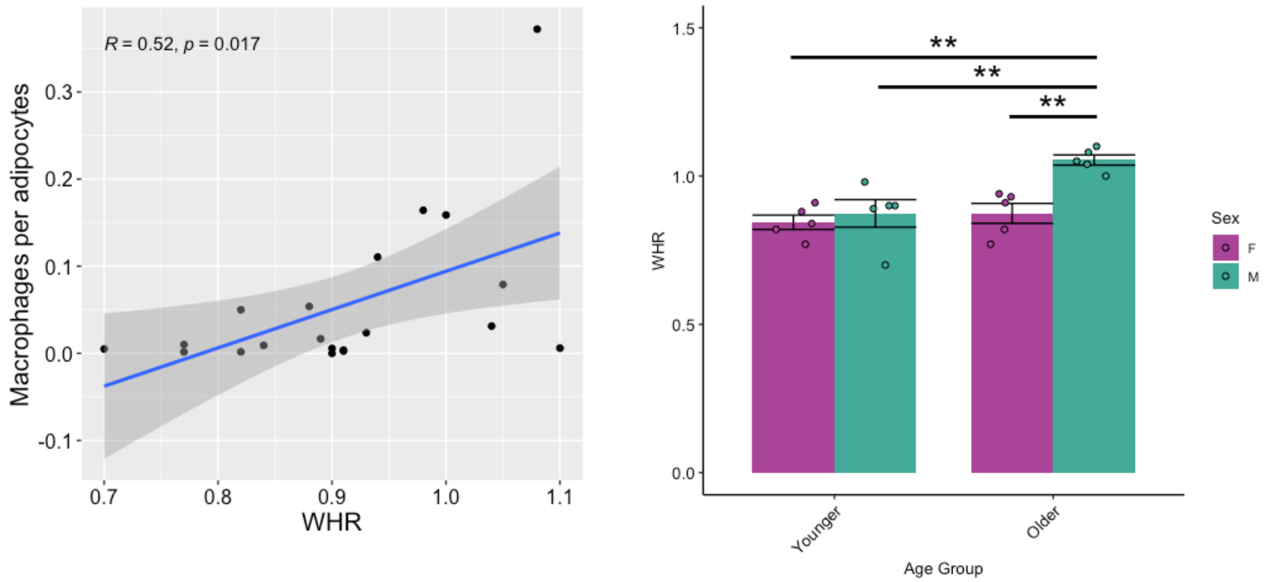


C)

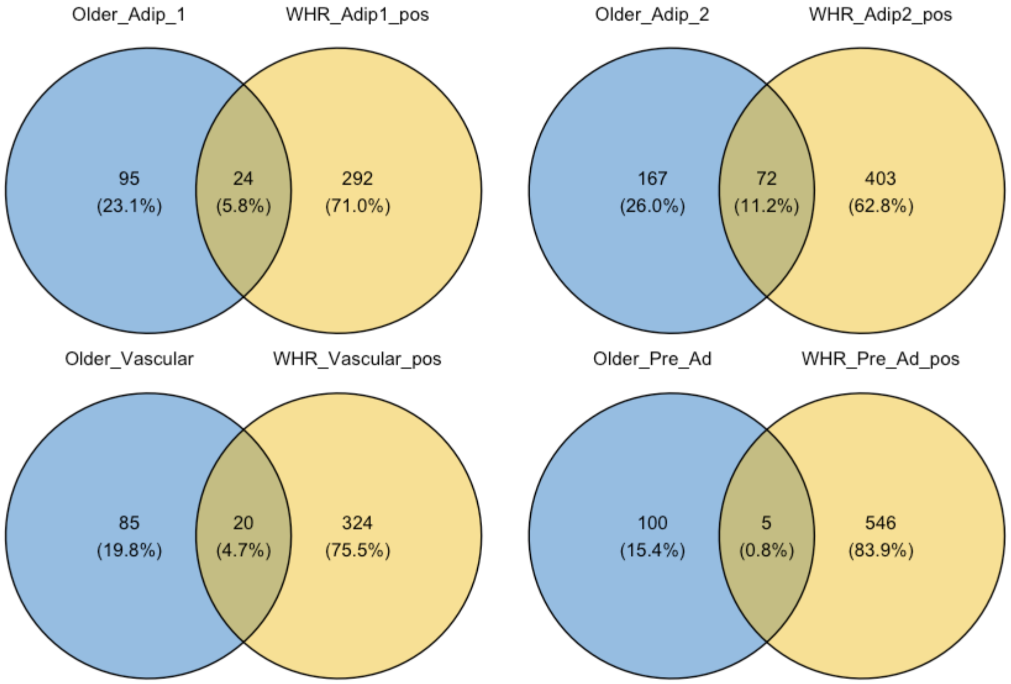


**Supplementary Figure 5**

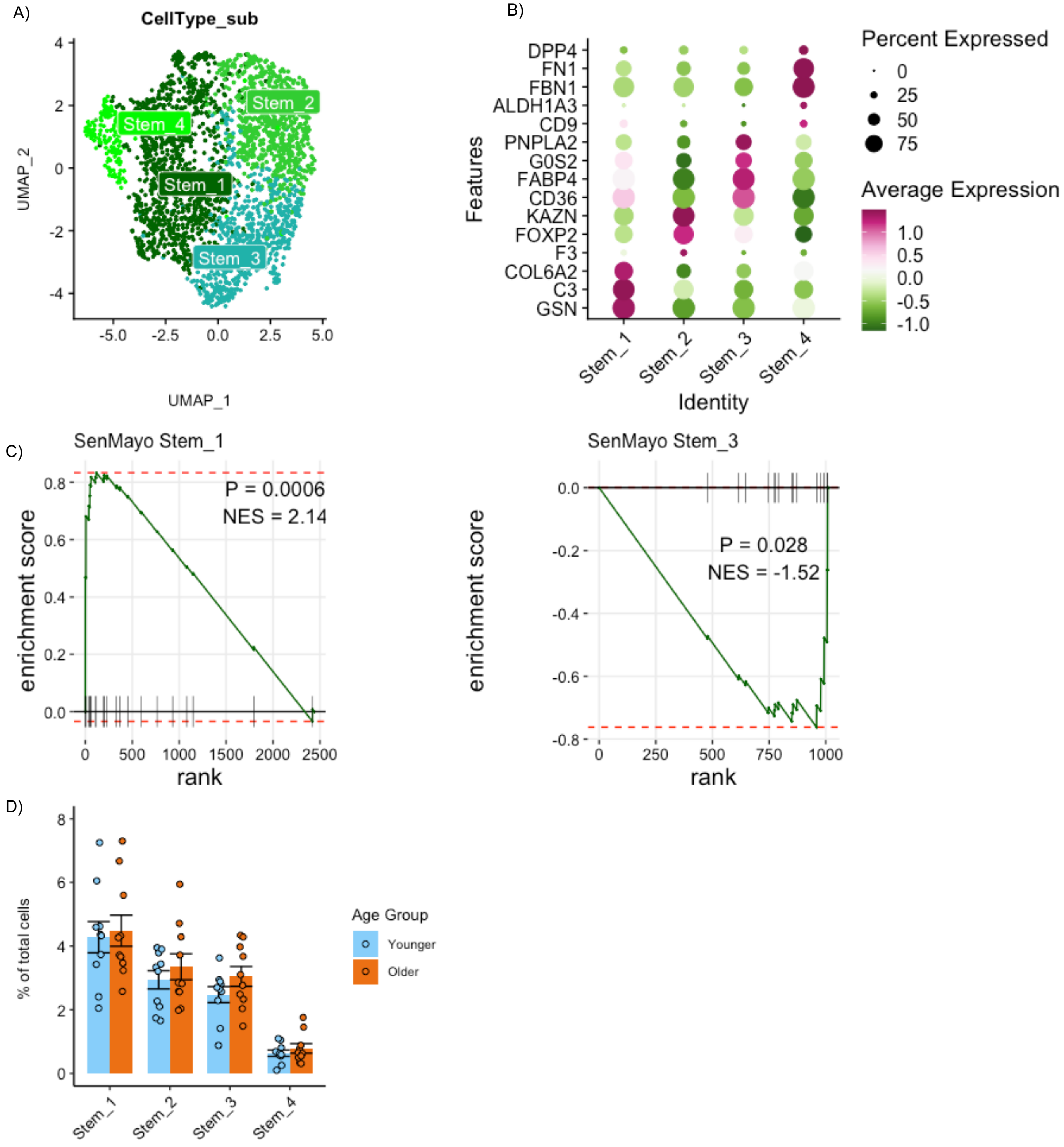


**Supplementary Figure 6**
